# The functions of DNA methylation by CcrM in *Caulobacter crescentus*: a global approach

**DOI:** 10.1093/nar/gkt1352

**Published:** 2014-01-07

**Authors:** Diego Gonzalez, Jennifer B. Kozdon, Harley H. McAdams, Lucy Shapiro, Justine Collier

**Affiliations:** ^1^Department of Fundamental Microbiology, University of Lausanne, Lausanne, CH 1015, Switzerland, ^2^Department of Developmental Biology, Stanford University, CA 94305, USA and ^3^Department of Chemistry, Stanford University, CA 94305, USA

## Abstract

DNA methylation is involved in a diversity of processes in bacteria, including maintenance of genome integrity and regulation of gene expression. Here, using *Caulobacter crescentus* as a model, we exploit genome-wide experimental methods to uncover the functions of CcrM, a DNA methyltransferase conserved in most *Alphaproteobacteria*. Using single molecule sequencing, we provide evidence that most CcrM target motifs (GANTC) switch from a fully methylated to a hemi-methylated state when they are replicated, and back to a fully methylated state at the onset of cell division. We show that DNA methylation by CcrM is not required for the control of the initiation of chromosome replication or for DNA mismatch repair. By contrast, our transcriptome analysis shows that >10% of the genes are misexpressed in cells lacking or constitutively over-expressing CcrM. Strikingly, GANTC methylation is needed for the efficient transcription of dozens of genes that are essential for cell cycle progression, in particular for DNA metabolism and cell division. Many of them are controlled by promoters methylated by CcrM and co-regulated by other global cell cycle regulators, demonstrating an extensive cross talk between DNA methylation and the complex regulatory network that controls the cell cycle of *C. crescentus* and, presumably, of many other *Alphaproteobacteria*.

## INTRODUCTION

Methylated bases can be found in the genomes of organisms from all three domains of life as well as in the genome of viruses (1–4). Aberrant cytosine methylation is associated with a variety of human diseases, including neurospychiatric disorders and cancers, exemplifying the critical functions of cytosine methylation, largely through its effects on the regulation of gene expression, in higher metazoans (5). The functions of DNA methylation have been poorly investigated for most of the bacterial kingdom (6). Bacterial DNA methyltransferases are mostly associated with endonucleases in restriction-modification systems, which are generally considered as a defense mechanism that bacteria use to identify and destroy differentially methylated foreign DNA (7–9). A number of ‘orphan’ DNA methyltransferases that are not associated with a cognate endonuclease have also been identified (7,10–12). The best studied examples are the DNA adenine methyltransferases Dam and CcrM. Dam methylates 5′-GATC-3′ (hereafter called GATC) motifs in the genomes of a subset of *Gammaproteobacteria* (13), whereas CcrM methylates 5′-GANTC-3′ (hereafter called GANTC) motifs in the genomes of many *Alphaproteobacteria*.

Dam has pleiotropic functions in *Gammaproteobacteria*: it is involved in the control of the initiation of chromosome replication (14–16), in the DNA mismatch repair (MMR) process (17,18) and in the regulation of gene expression (7,10). DNA microarrays were used to compare global transcription patterns in wild type, Δ*dam* and Dam-over-expressing *Escherichia coli* cells (19–22). These studies revealed that hundreds of genes were misregulated in these two mutant strains. Similar effects were observed in *Salmonella enterica* (23). In addition, detailed studies in *E. coli* and *S. enterica* have led to the identification of a number of genes that show a Dam-dependent transcriptional phase variation, creating heterogeneity of expression rates within the bacterial population (11,24). Such mechanisms of regulation often involve transcription factors that compete with Dam for DNA binding and whose binding affinity is sensitive to the methylation state of the DNA at or near their binding site. In some cases, the presence of one or more under-methylated GATC motifs in a promoter is an indication that it is regulated by such a bistable epigenetic switch, although there also exists under-methylated GATC motifs that are not part of epigenetic mechanisms regulating gene expression (25,26). Dam is to date the only DNA adenine methyltransferase whose global effects on gene expression have been studied in bacteria.

**Figure 1.**
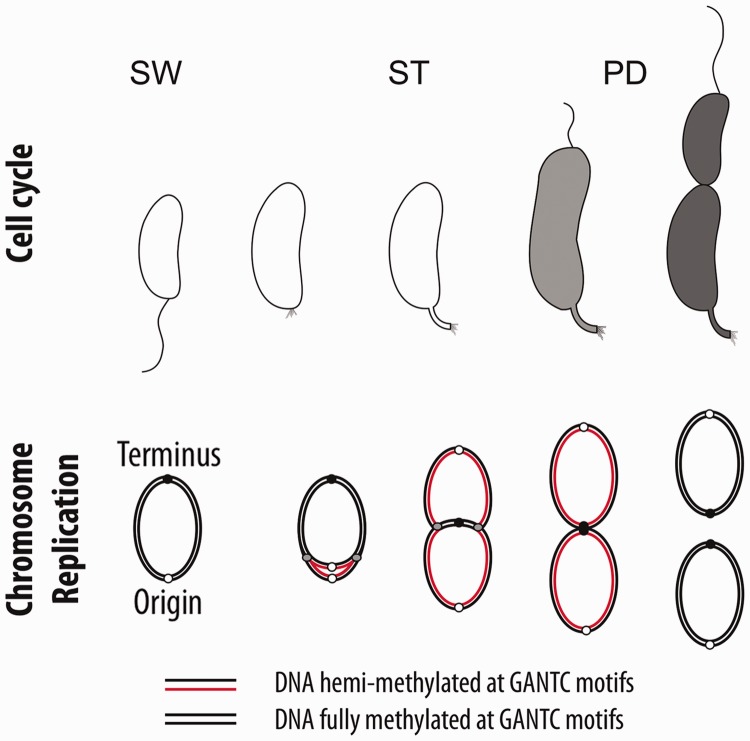
CcrM is a cell cycle–regulated DNA methyltransferase in *C. crescentus*. Upper panel: schematic of the *C. crescentus* cell cycle, showing the differentiation of a swarmer cell (SW) into a stalked cell (ST), which then turns into a pre-divisional cell (PD). Lower panel: schematic showing the changes in the methylation state of GANTC motifs on the chromosome as a function of chromosome replication and cell cycle progression.

The cell cycle–regulated methyltransferase CcrM was first identified in *Caulobacter crescentus*, an *Alphaproteobacterium* that divides asymmetrically into two morphologically different progeny cells: the daughter stalked cell that immediately initiates DNA replication and cell division and the daughter swarmer cell that first differentiates into a stalked cell before replication and cell division (Figure 1) (27,28). This bacterium initiates the replication of its chromosome only once per cell cycle and specifically in the stalked progeny or during the swarmer-to-stalked cell transition (29,30). The expression of CcrM is restricted to the late predivisional stage near the end of chromosome replication, which implies that GANTC motifs on the chromosome are supposedly fully methylated (methylated on both DNA strands) in swarmer cells and then become hemi-methylated (methylated on one strand only) on DNA replication in stalked cells, and stay so until the end of the replication of the chromosome in pre-divisional cells (Figure 1) (30–32). Maintaining the chromosome constitutively fully methylated by over-expressing CcrM leads to morphological and cell cycle–related defects in *C. crescentus*, suggesting that the temporal regulation of the transition from the fully methylated to the hemi-methylated state has biological importance (31,33). In *C. crescentus,* the methylation state of GANTC motifs has a direct effect on the transcription of at least four genes: the *ctrA* and *dnaA* genes encoding master regulators of the cell cycle (34,35), the *ftsZ* and *mipZ* genes required for cell division (36) and, most probably, the *ccrM* gene itself (37). The transition from the fully methylated to the hemi-methylated state of a GANTC motif located in *cis* is sufficient to cause a small but significant change in the transcriptional activity of the *ctrA*, *dnaA* and *ftsZ* promoters. This supports a model according to which the change in GANTC methylation state during DNA replication might function to co-ordinate gene expression changes with the progression of the replication fork, setting the mechanical basis for the regulation of the timing of multiple cell cycle events (32,34,35). We recently showed that an increase in *ftsZ* transcription is sufficient to restore the viability of Δ*ccrM* cells in rich medium, demonstrating that the direct activation of *ftsZ* transcription by DNA methylation is one of the most important functions of CcrM in *C. crescentus* (36). These rescued cells still exhibited a residual phenotype. For example, cells were elongated and straighter than wild-type cells and had shorter stalks. These observations suggested that more genes are probably regulated by CcrM-dependent methylation in *C. crescentus*. Interestingly, the *ccrM* gene is not essential anymore for the viability of *C. crescentus* when cells are cultivated in slow-growing conditions, such as minimal medium: these cells are nevertheless slightly elongated and grow slightly more slowly than wild-type cells (36). The physiological importance of GANTC methylation is probably not restricted to *C. crescentus*, as the homolog of *ccrM* was shown to be essential for viability in fast-growing cultures of each of the species where this was tested (33,36,38–40).

Here, we assess the global influence of GANTC methylation on transcription profiles in *C. crescentus* by analyzing the transcriptome of a Δ*ccrM* strain and of a strain in which CcrM is constitutively expressed so that the chromosome remains in the fully methylated state throughout the cell cycle. We show that hundreds of genes are differentially expressed in both mutant strains compared with the wild type. Genes whose transcription could directly be affected by GANTC methylation are predicted on the basis of their differential expression in the mutant strains and through bioinformatic analysis of GANTC conservation in promoters. Our findings reveal a strong link between CcrM-dependent methylation and cell cycle control in *C. crescentus* that is also likely to exist in many other *Alphaproteobacteria*.

## MATERIALS AND METHODS

### Phylogenetic analysis

NCBI whole bacterial proteomes were searched for proteins with homology to the CcrM protein from *C. crescentus* strain CB15 (blastp e-value < 1e-10) (41); one strain was selected per genus and the protein with the lowest blastp e-value was used for each individual strain. On the basis of a MUSCLE alignment of the sequences (42,43), the subtype β N6-adenine-methyltransferases were selected, using the motif IV [DPPY ≠ SPP(Y/F)] and the motif X [T(Q/E/D)KP ≠ AXFP] to discriminate between N6-methyltransferases and N4-methyltransferases (44). A tree was calculated with FastTree (45,46) using the WAG matrix for amino-acid substitutions to get a general idea of the topology (Supplementary Figure S1B). Our final set comprised all sequences of CcrM-homologs, which had a conserved C-terminal domain, i.e. sequences from *Alphaproteobacteria*, *Helicobacter* and some *Chloroflexi*, with the addition of *Haemophilus influenzae* HinfI [from REBASE (8)]; on the basis of the FastTree tree, two sequences were added as outgroups (from *Streptococcus mitis* and *Stigmatella aurantiaca*). A new alignment was made with MUSCLE and curated with GBlocks 0.91b (47) with default options and gaps allowed at all positions. The final trees were calculated on the basis of the curated alignment using RAxML (48) or MrBayes version 3.2.1 with 200 000 iterations and 2 × 4 chains (49,50) with the WAG matrix for amino-acid substitution and the Gamma model of substitution rate heterogeneity. Branches with support values <0.5 were left unresolved (Supplementary Figure S1C).

### Distribution of pentanucleotides in the genomes of *Alphaproteobacteria*

Bacterial genome sequences were downloaded from the NCBI repository. GANTC motifs and other pentanucleotides were counted in the complete genome sequences (.fna file), in the protein coding sequences (.ffn file) and in the RNA coding sequences (.frn file). The number of GANTC motifs and other pentanucleotides in intergenic sequences was calculated as follows: number in the genome − (number in protein coding sequences + number in RNA coding sequences). The frequency of each nucleotide in each complete genome sequence was calculated as the ratio between the number of each nucleotide and the total number of nucleotides of the whole genome. The expected number of a given pentanucleotide with a central variable N in a genome was calculated as freq(A)*freq(C)*freq(G)*freq(A)*(genome length), where freq(A) is the frequency of As in the genome, for example, and ‘genome length’ is the total number of nucleotides in the considered genome. The expected number of GANTC motifs and other pentanucleotides in protein coding, RNA coding and non-coding sequences was calculated as (subsequence length/genome sequence length)*(total pentanucleotide number), where ‘subsequence length’ is the length of the protein coding, RNA coding and non-coding sequences, respectively, and the total pentanucleotide number is the total number of a given pentanucleotide found in the considered genome. As ‘Other *Proteobacteria*’ we used the genomes of the following: *Neisseria gonorrhoeae FA1090*, *Burkholderia mallei ATCC 23344*, *Nitrosomonas europaea ATCC 19718*, *Myxococcus xanthus DK1622*, *Bdellovibrio bacteriovorus HD100*, *Desulfovibrio vulgaris DP4*, *Helicobacter pylori F32*, *Nautilia profundicola AmH*, *Sulfurospirillum deleyianum DSM 6946*, *E. coli str. K-12 substr. DH10B*, *Pseudomonas aeruginosa PAO1*, *Vibrio cholerae O1 biovar El Tor str. N16961*.

### Growth conditions and bacterial strains

Three *C. crescentus* strains were used in this study: (i) Strain NA1000 (CB15N) is a synchronizable derivative of the wild-type CB15 strain (51,52); (ii) Strain JC362 (53) is a derivative of the NA1000 strain expressing a second copy of the *ccrM* gene under the control of the endogenous *lacZ* promoter (P*lac*::*ccrM*), which is constitutively active (31); and (iii) Strain JC1149 (36) is a derivative of the NA1000 strain with a deletion of the *ccrM* gene (Δ*ccrM*). Cells were cultivated in M2G minimal medium at 28^°^C. Experiments were all performed using unsynchronized cell populations because well-synchronized JC1149 populations could not be isolated, probably due to the slightly abnormal morphology of the Δ*ccrM* cells (36).

### Single molecule real time sequencing analysis

Genomic DNA was extracted from cells cultivated to exponential phase using the Qiagen Puregene Yeast/Bact DNA extraction kit with a second ethanol precipitation step after resuspension; the DNA was finally dissolved in 10 mM Tris–HCl, pH 8. For each DNA sample, Single Molecule Real Time (SMRT) Bell libraries with a 2-kb insert size were sequenced in 6 SMRT cells and the data were analyzed with tools provided in the Pacific Biosciences SMRT portal using the NC_011916 NA1000 genome (NCBI) as a reference. Mean depth coverage was 182 for strain NA1000, 187 for strain JC362 and 37 for strain JC1149. SMRT confidence scores, defined as -10log(p-value), for methylated adenines were typically high in GANTC motifs for the NA1000 and JC362 strains (median is 115 and 145, respectively) and low for the JC1149 strain (median is 3). For adenines in all GANTC motifs in the chromosome, the inter-pulse duration (IPD) ratio was calculated by dividing the average measured IPD for the nucleotide by the IPD value predicted in an *in silico* model for non-methylated DNA. IPD ratios were calculated for all adenines in GANTC contexts on both DNA strands. We used the average IPD ratio for adenines at GANTC motifs in the chromosome of strain JC362 to approximate the value for adenines fully methylated in chromosomes in the whole population (=7.68); the theoretical average IPD ratio for adenines hemi-methylated in chromosomes in the whole population would then be (7.68 + 1)/2 = 4.34. An IPD of 2.4, corresponding to an adenine fully methylated in chromosomes from 20% of the population or hemi-methylated in 40% of the population was chosen as a threshold to consider that a GANTC motif was under-methylated.

### Preparation of RNA samples for transcriptome or qRT-PCR analysis

All strains were cultivated in triplicates and independent cultures (NA1000 versus JC1149) or independent biological samples (NA1000 versus JC362) were used for subsequent transcriptome and quantitative real-time polymerase chain reaction (qRT-PCR) analysis. Two milliliters of cell culture aliquots were pelleted, frozen in liquid nitrogen and stored at −80°C. Pellets were homogenized in 1 ml of Trizol reagent and incubated at 65°C for 10 min. Two hundred microliters of chloroform were added and, after 3 min at room temperature, the extracts were centrifuged for 15 min at maximum speed. Five hundred fifty microliters of 100% ethanol were added to 550 µl of aqueous phase from the Trizol treatment and RNA was purified using the Ambion PureLink RNA mini-kit (standard protocol without the lysis step). RNA was eluted in 40 µl of H_2_O, treated in a 60-µl reaction with 10 µl of Invitrogen DNase I Amp Grade for 30 min at room temperature.

### Transcriptome analysis

Custom microarrays were designed by Nimblegen using *C. crescentus* NA1000 protein and small RNA coding sequences. When possible, three different probes of ∼60 bp were designed per gene. Microarrays had a 4-plex format (four sub-arrays per chip); each probe was printed in randomly positioned triplicates in each of the sub-arrays. One sub-array was used per biological sample. RNA samples were prepared as described above and 6 µl of EDTA 25 mM (Invitrogen) were added for a 10-min incubation at 65°C to inactivate the DNase I. Sixty microliters of lysis buffer from the PureLink RNA kit with 1% 2-mercaptoethanol and 60 µl of 100% ethanol were added to the reaction and the RNA was purified again using the PureLink standard protocol (without the lysis step). The RNA was resuspended in 32 µl, from which 2 µl were used for quality check via Bioanalyzer and quantification with Nanodrop. cDNA was synthetized from 5 µg of RNA using the Roche double-stranded cDNA synthesis system (cat. 11′117′831′001) and the protocol described in the ‘Roche cDNA Synthesis System for use with NimbleGen Gene Expression Microarrays’ technical note. Promega random hexamers at the same concentration were used instead of the oligo(dT)_15_. The double-stranded cDNA was precipitated with isopropanol, purified and resuspended in 40 µl according to the protocol. The whole purified double-stranded cDNA (0.5–1 µg cDNA) was used as input for the standard Klenow-based NimbleGen Cy3 labelling protocol. Two micrograms of labelled cDNA were hybridized 16 h in one sub-array using the standard Nimblegen protocol for assembly, hybridization and washing. Arrays were scanned in an Agilent High-Resolution Microarray scanner at 2 µm resolution. Normalized intensities were extracted from the pictures with the Nimblescan 2.6 software using the standard workflow and default options. The Robust Multi-array Average (RMA) module of the software was used to normalize the intensity values for all chips. The statistical analysis ‘mutant versus wild type’ was performed with the R limma package on the basis of the intensity values of all biological replicates for the mutant and wild-type strains. Genes with an adjusted *P* < 0.01 were considered significantly up- or downregulated. The logFC value given by the limma function was used as the log-ratio shown in Tables.

Flow cytometry analysis, methods to determine spontaneous mutation rates and methods for qRT-PCR analysis are described in Supplementary Information.

## RESULTS

### CcrM homologs are conserved in most *Alphaproteobacteria*

**Figure 2.**
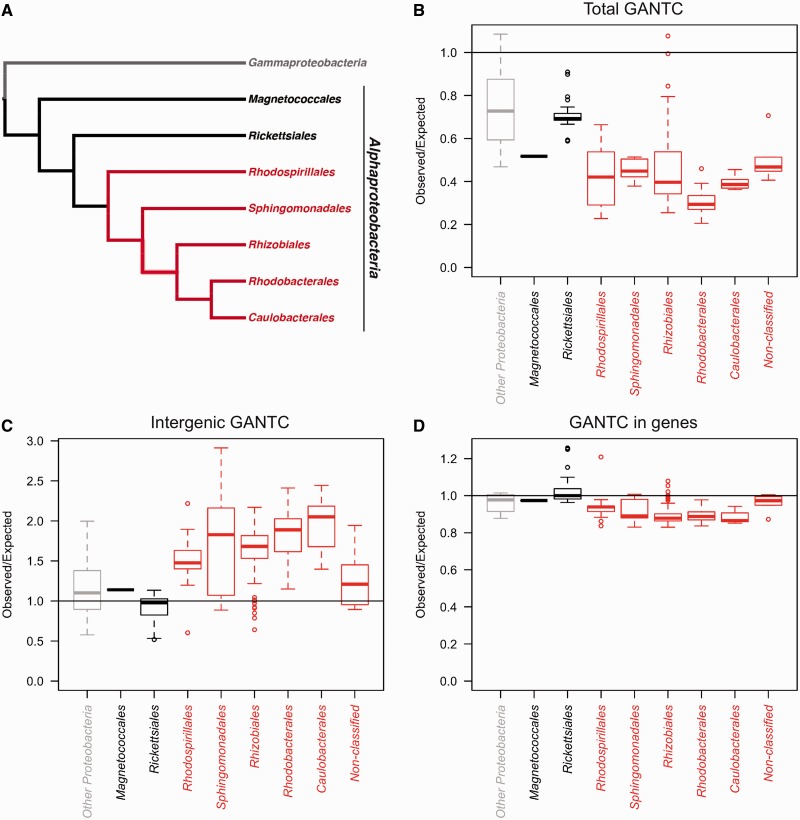
Conservation of CcrM in *Alphaproteobacteria* and distribution of GANTC motifs in their genomes. (**A**) Schematic showing the conservation of CcrM in different orders of *Alphaproteobacteria*. Panels (B), (C) and (D) show the observed-over-expected GANTC ratios using the whole genome (**B**), the intergenic sequences (**C**) and the coding sequences (**D**) of sequenced bacterial species from different orders of *Alphaproteobacteria*. ‘Other *Proteobacteria*’ correspond to a control of 10 mixed other *Proteobacteria*. The expected number of motifs was calculated for each genome taking the nucleotide composition into account. The limits of the box represent the first and third quartiles and the bold line the median of the distribution. In the whole figure, orders of bacterial species containing a CcrM homolog are indicated in red.

The functions of the CcrM family of N6-adenine methyltransferases have been studied in a limited number of *Alphaproteobacteria*, including *C. crescentus*, *Agrobacterium tumefaciens*, *Rhizobium meliloti* and *Brucella abortus* (33,38–40). To assess the phylogenetic conservation of CcrM homologs beyond these species and more thoroughly than before (54), we searched for proteins with a significant similarity to the *C. crescentus* CcrM protein in all available bacterial proteomes (Supplementary Table S1). We found that all *Alphaproteobacteria* except *Rickettsiales* and *Magnetococcales* (55) had a CcrM homolog with a high similarity score (Figure 2A). As the target recognition domain of CcrM is conserved in each of these proteins, these enzymes most likely also methylate the adenine in GANTC motifs. The only exceptions were the bacteria belonging to the SAR11 clade (e.g. *Pelagibacter ubique* and related species) that do have a CcrM homolog although they have sometimes been classified as *Rickettsiales* (56,57), and the cicada endosymbiont *Candidatus Hodgkinia cicadicola* Dsem (*Rhizobiale*) whose genome does not encode a CcrM homolog. Outside *Alphaproteobacteria*, the genomes of sporadic species and clades across the phylogeny encode a protein closely related to CcrM. *ccrM* homologs were only found in close proximity to an endonuclease gene on a chromosome in several *Epsilonproteobacteria* or *Gammaproteobacteria* (Supplementary Table S2), suggesting that CcrM homologs are orphan N6-adenine methyltransferases in all *Alphaproteobacteria*.

A phylogenetic tree was built using the sequences of proteins with a high similarity to the *C. crescentus* CcrM protein, belonging to the β-class of N6-adenine methyltransferases and comprising a conserved additional C-terminal domain specific to the CcrM family (Supplementary Figure S1). The most parsimonious scenario compatible with the phylogenetic tree is that the gene encoding the ancestor of the CcrM homolog entered the genome of an ancestral *Alphaproteobacterium* after the divergence of *Magnetoccocales* and *Rickettsiales* and that it was thereafter transmitted vertically to the descendent species (Figure 2A). The pervasiveness of vertical transmission suggests that the CcrM homolog is part of the core genome and plays a critical physiological role in the large and diverse terminal clade of the alphaproteobacterial tree.

### GANTC motifs are under-represented in the genomes of *Alphaproteobacteria* but over-represented in intergenic regions

Previous studies indicated that GANTC motifs are less frequent in the genome of *C. crescentus* than expected in a random sequence of nucleotides and that these motifs are over-represented in intergenic regions; these biases might be connected to the biological role that DNA methylation plays in controlling transcription or other cellular processes in *C. crescentus* (35,58,59). To explore this possibility more systematically, we analyzed the frequency and distribution of GANTC motifs in all available genomes of *Alphaproteobacteria* and of a control set of other *Proteobacteria*. We found that GANTC motifs were on average at least 2-fold less frequent than expected in the genomes of all orders of *Alphaproteobacteria,* including *Magnetococcales* but excluding *Rickettsiales* (Figure 2B). In the group of other *Proteobacteria*, this bias was largely attenuated. Notably, we also observed that GANTC motifs showed a distribution bias between protein coding and intergenic sequences in each order comprising bacterial species encoding a CcrM homolog (Figure 2C and D), being on average >1.5-fold over-represented in intergenic regions and slightly under-represented in coding regions. These biases were largely attenuated in the group of other *Proteobacteria* lacking a CcrM homolog. Overall, these findings suggest that selective pressure limits the overall frequency of GANTC motifs in protein coding sequences on the genomes of bacteria encoding a CcrM homolog, whereas they are tolerated or favoured in intergenic sequences.

To check whether these biases were specific to GANTC motifs, we also analyzed the frequency and distribution of the 24 pentanucleotides of the same structure as GANTC (each of the four bases present one time, one central variable N) (Supplementary Figure S2). Surprisingly, another one of these pentanucleotides, CTNAG, presented similar strong frequency biases, not only in *Alphaproteobacteria* encoding CcrM homologs, but also in the control group of other *Proteobacteria* (Supplementary Figure S3). Thus, a bias in the frequency and distribution of pentanucleotides of that structure can be found in bacterial genomes independently of their capacity to be methylated by CcrM homologs, indicating that the biased distribution of GANTC motifs in the genomes of CcrM-encoding *Alphaproteobacteria* might also be explained by other factors.

### The methylation of GANTC motifs in the genome of *C. crescentus* is strictly dependent on CcrM

**Figure 3.**
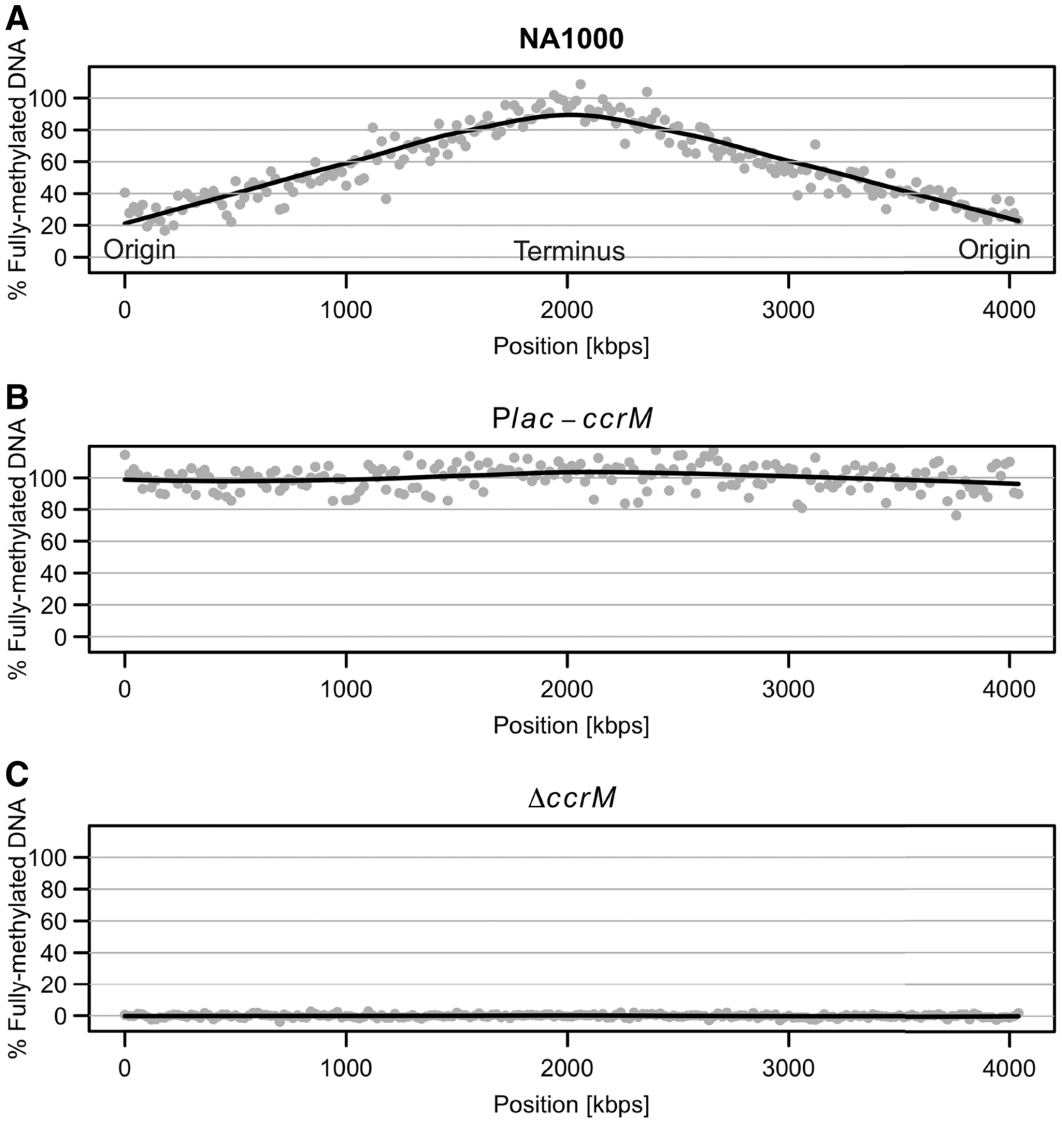
CcrM-dependent methylation of GANTC motifs in the genome of *C. crescentus*. Predicted methylation state of the DNA, as % of the chromosomal population being fully methylated at a particular locus, along the chromosome of *C. crescentus* (**A**) NA1000, (**B**) P*lac::ccrM* [JC362], (**C**) Δ*ccrM* [JC1149]). We used the SMRT portal to detect methylated motifs in the chromosome of *C. crescentus* and extracted the IPD ratios for adenines in all GANTC motifs (both strands). We then calculated the average IPD ratio in 20 kb windows along the chromosome and plotted the values according to the chromosomal co-ordinates (the origin of replication is situated at the junction between 4000 kb and 1 kb). The average IPD ratio for strain JC362 was equaled to 100% and the theoretical value for hemi-methylated DNA [(value for fully methylated DNA + 1)/2] as 0%; the curve was fitted with the R loess function.

The temporal regulation of the methylation of GANTC motifs seems important in a variety of *Alphaproteobacteria*, since cells that constitutively express homologs of CcrM often appear abnormal (31,34,39,40,60). A system of DNA methylation probes distributed at a dozen positions along the *C. crescentus* chromosome previously suggested that newly replicated GANTC motifs stay hemi-methylated until the pre-divisional stage of the *C. crescentus* cell cycle, independently of the moment when they got replicated, due to the strict temporal regulation of CcrM concentration during the cell cycle (30,31). According to this model, GANTC motifs located close to the chromosomal origin should stay hemi-methylated for a longer period during the cell cycle than motifs located close to the chromosomal terminus, as they are replicated earlier during the cell cycle. To confirm this model for each of the GANTC motifs on the *C. crescentus* chromosome, we made use of the recently developed SMRT sequencing technology of PacBio on genomic DNA extracted from wild-type, Δ*ccrM* (36) or CcrM-over-expressing (31) cells cultivated to exponential phase. This technology measures the time needed for a DNA polymerase to incorporate a nucleotide on a template DNA (IPD), which varies depending on the methylation state of the nucleotide found on the template DNA (61). This method is powerful at detecting N6-methyladenines on bacterial chromosomes (62,63) and we used it to estimate the proportion of cells in the bacterial population in which a given GANTC motif is methylated on either strand. We observed a positive correlation between the proximity of a GANTC motif to the chromosomal origin and its probability to be hemi-methylated in a mixed population of the wild-type strain (Figure 3A). We estimated that motifs located close to the chromosomal origin were, on average, hemi-methylated in ∼80% of the cells, while sites located close to the chromosomal terminus were hemi-methylated in <10% of the cells. In the Δ*ccrM* population, we found that none of the GANTC motifs were fully methylated (Figure 3C), confirming that the methylation of each GANTC motif on the *C. crescentus* chromosome is dependent on CcrM. In the CcrM-over-expressing population, we observed that nearly all GANTC motifs were fully methylated in the whole population (Figure 3B), showing that the fully methylated to hemi-methylated switch that takes place for most of the GANTC motifs on the *C. crescentus* chromosome is dependent on the precise temporal regulation of the concentration of CcrM during the cell cycle. These results confirmed, at the genomic scale and in a quantitative manner, the model previously proposed by Zweiger *et al.* for most GANTC motifs (31).

Interestingly, through our SMRT sequencing analysis, we found 35 exceptions to this rule: 24 GANTC motifs were strongly under-methylated and 11 additional GANTC motifs were asymmetrically methylated (one strand being more often under-methylated than the other one) in a population of unsynchronized wild-type *C. crescentus* cells (Supplementary Table S3). A recent study using a synchronized population of *C. crescentus* cells demonstrated that most of these motifs remain under-methylated throughout the whole cell cycle (64). Here, we show that only three of these motifs remained under-methylated in cells over-expressing CcrM, while the other 32 GANTC motifs were efficiently methylated on both DNA strands.

Overall, these results demonstrate that the Δ*ccrM* and CcrM-over-expressing strains are accurate tools for exploring the functions of GANTC methylation in *C. crescentus* and the functions of the methylation switch that occurs at most GANTC motifs over the course of the cell cycle of *C. crescentus*.

### Control of chromosome replication and spontaneous mutation rate in the absence of GANTC methylation in *C. crescentus*

The orphan adenine methyltransferase Dam was shown to be required for the replication of one of the two chromosomes in *V. cholerae* (15,16) and to be involved in the control of the initiation of chromosome replication in *E. coli* (14,65). Several GANTC motifs can be found in the origin of replication of *C. crescentus* and of other closely related species (66) and it was previously shown that cells over-expressing CcrM accumulate additional chromosomal copies (31). We thus hypothesized that CcrM might be required for the regulation of chromosome replication in *C. crescentus*, ensuring that it occurs only once per cell cycle (30,31). To test this hypothesis, we used flow cytometry to compare the DNA content of cells in wild-type and Δ*ccrM* populations treated with rifampicin. Rifampicin blocks the initiation of DNA replication, but not the elongation of DNA replication, so that wild-type *C. crescentus* cells accumulate either one or two complete chromosomes after rifampicin treatment (Supplementary Figure S4A). We observed that Δ*ccrM* populations cultivated in minimal medium had a DNA content profile similar to wild-type populations (Supplementary Figure S4B). We concluded that CcrM is neither required for the control of the initiation of chromosome replication, nor for the correct segregation of replicating chromosomes before cell division, unlike Dam in several enterobacteria (7,14).

In addition to its regulatory roles, Dam plays an important role in DNA MMR in a subset of *Gammaproteobacteria* (17,18): the hemi-methylated state of GATC motifs behind the replication fork provides critical information to identify the newly replicated strand, more likely to contain errors that need to be repaired (18). This raised the possibility that the methylation of GANTC motifs by CcrM may similarly be used for DNA repair in *C. crescentus*. We therefore compared the spontaneous mutation rates of the wild-type and Δ*ccrM* strains by calculating the frequency of rifampicin resistant mutants arising in each population and found that they were similar (Supplementary Figure S5). This result shows that methylation of GANTC motifs by CcrM is not needed for the MMR mechanism in *C. crescentus*.

Cumulatively, these observations indicate that CcrM in *Alphaproteobacteria* does not share the most conserved functions of Dam in *Gammaproteobacteria*. This is consistent with the different evolutionary histories of the two proteins. Furthermore, we provide evidence below that the impact of CcrM-dependent methylation on the regulation of gene expression in *C. crescentus* also differs from the known impact of Dam-dependent methylation in *E. coli* (19–21).

### Alterations in gene expression in the absence of GANTC methylation in C. *crescentus*

**Figure 4.**
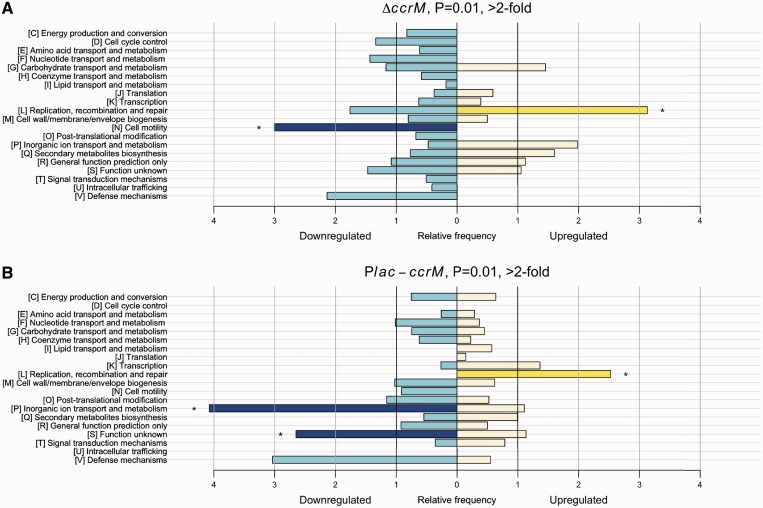
Relative frequency of functional categories among genes found as strongly misregulated during the transcriptome analysis. Genes whose expression changed > 2-fold in the Δ*ccrM* (**A**) and the CcrM-over-expressing (**B**) strains were selected. Blue and yellow bars represent the set of genes whose expression is downregulated and upregulated, respectively, in the corresponding strain. Stars, dark blue and bright yellow colours indicate a significant over-representation (*P* < 0.05, Fisher’s exact test).

To evaluate the impact of CcrM-dependent DNA methylation on global gene expression profiles in *C. crescentus*, we used custom-made DNA microarrays to compare the transcription levels in wild-type and Δ*ccrM* populations cultivated to exponential phase in minimal medium. We found that out of the 3932 genes in the *C. crescentus* NA1000 genome for which probes were designed, the expression levels of 388 genes were significantly (corrected Student’s test *P* < 0.01) changed in the Δ*ccrM* strain compared with the wild-type strain, with 152 of the 388 genes being affected >2-fold (Supplementary Table S4). For 17 genes, the effects were verified by qRT-PCR and were essentially consistent with the microarray results (Supplementary Figure S6 and Table 1). We classified each gene strongly misregulated in Δ*ccrM* cells according to the known or predicted function of the protein that it encodes (NCBI single letter COG) (Figure 4A). We found that genes encoding proteins involved in DNA replication, recombination or repair were significantly enriched among the genes that were upregulated in the absence of CcrM, while genes encoding proteins involved in cell motility were significantly enriched among the genes that were downregulated in the absence of CcrM (Figure 4A).

**Table 1.** Essential genes containing GANTC motifs in their promoter region and significantly misregulated in Δ*ccrM* cells compared with wild-type *C. crescentus* cells

Gene name	Known or predicted function	Log-ratio MA	Log-ratio qRT-PCR	CtrA regulon	DnaA regulon	GcrA regulon	GANTC conservation
Essential genes repressed in Δ*ccrM*							
* CCNA_00390*	ADP-heptose—LPS heptosyltransferase	−0.85	−0.72	No	No	No	4
* CCNA_01104*	Glycosyltransferase	−0.77	ND	No	No	No	2
* CCNA_01275*	Methylenetetrahydrofolate dehydrogenase (NADP+)/methenyltetrahydrofolate cyclohydrolase	−0.84	ND	No	No	No	1
* CCNA_01450*	Single-stranded DNA-specific exonuclease RecJ	−1.03	−1.20	No	No	Yes	4
* CCNA_01535*	Single-stranded DNA binding protein	−0.56	ND	No	Yes	No	2
* CCNA_01590*	NAD-dependent DNA ligase	−1.23	ND	No	No	No	3
* CCNA_01651*	DNA gyrase subunit A	−1.14	−1.01	No	No	Yes	5
* CCNA_01776*	Ribonuclease D	−0.68	−0.92	No	No	No	5
* CCNA_02052*	Topoisomerase IV subunit B	−1.97	ND	No	No	Yes	3
* CCNA_02086*	Cell division protein FtsN	−1.09	ND	No	No	Yes	5
* CCNA_02127*	Oxacillin resistance-associated protein FmtC	−1.93	ND	No	No	Yes	2
* CCNA_02208*	Thymidylate synthase	−1.03	ND	No	No	No	3
* CCNA_02246*	Division plane positioning ATPase MipZ	−0.52 ^a^	−2.54	Yes	Yes	Yes	5
* CCNA_02256*	Mg2+ transporter MgtE	−0.83	−1.14	No	No	No	5
* CCNA_02389*	Glucosamine-1-phosphate acetyltransferase/ UDP-N-acetylglucosamine pyrophosphorylase	−0.87	−0.69	No	No	No	5
* CCNA_02623*	Cell division protein FtsZ	−1.44	−1.75	Yes	Yes	No	5
* CCNA_02679*	Hypothetical protein	−1.07	ND	No	No	No	3
* CCNA_03607*	Ribonucleoside-diphosphate reductase subunit alpha NrdA	−0.47	−0.77	No	Yes	No	5
Essential genes activated in Δ*ccrM*							
* CCNA_00379*	Thiol:disulfide interchange protein DsbA	0.69	0.53	No	No	No	5
* CCNA_00845*	Antitoxin protein RelB-1	0.82	ND	No	No	No	1
* CCNA_00893*	Transcriptional regulatory protein	0.53	ND	No	No	No	0
* CCNA_01946*	Tyrosyl-tRNA synthetase	0.89	0.28	No	No	No	4
* CCNA_02635*	Cell division protein FtsW	0.65	ND	Yes	No	No	2
* CCNA_03130*	Cell cycle response regulator CtrA	0.46	0.34	Yes	No	Yes	5
* CCNA_03471*	4-hydroxy-3-methylbut-2-enyl diphosphate reductase	0.74	0.23	No	No	No	4
* CCNA_03766*	16 S rRNA processing protein RimM	1.11	ND	No	No	No	3
* CCNA_03820*	Outer-membrane lipoproteins carrier protein	1.09	ND	No	No	No	3

Genes were considered essential according to (67). The log-ratios indicate the expression change observed using microarrays (MA) or qRT-PCR comparing wild-type and Δ*ccrM* cells. ND indicates that the log-ratio was not measured by qRT-PCR for that gene.

^a^Indicates that unspecific low-signal probes were unfortunately designed to detect *mipZ* by microarrays. The CtrA and DnaA direct regulons were described in (68) and (69), respectively. The GcrA regulon was described in (70). The ‘GANTC conservation’ column indicates the number of species in which a GANTC motif is found in the promoter region (200 bp upstream of their translational start codon) of homologous genes among five bacterial species closely related to *C. crescentus* (*C. crescentus*, *C. segnis*, *C. K31*, *Phenylobacter zucineum*, *Brevundimonas subvibrioides*).

**Figure 5.**
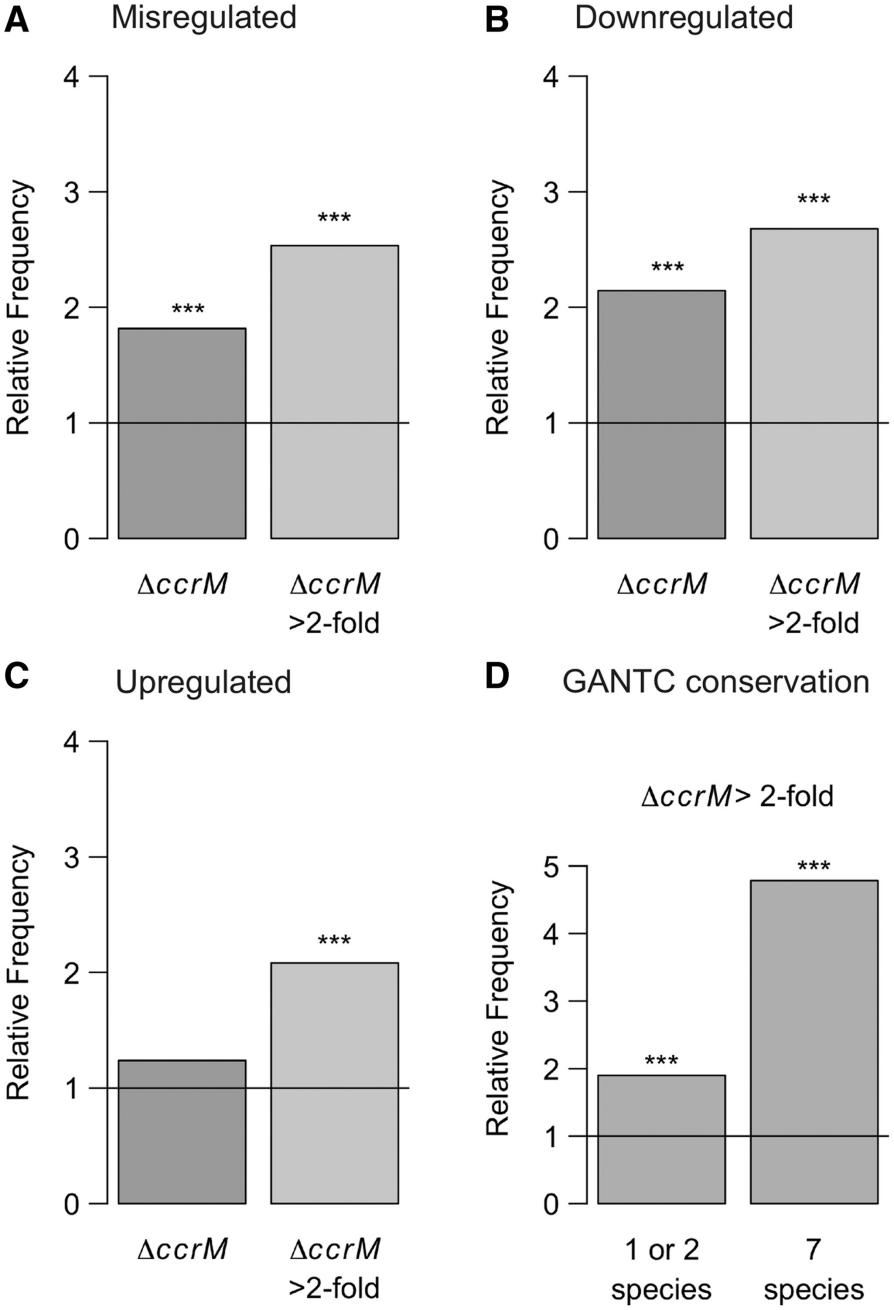
Conserved GANTC motifs are enriched upstream of genes significantly misregulated in the Δ*ccrM* strain. Frequency, relative to the entire genome, of genes whose promoter region (here, 200 bp upstream of their translational start codon) contains a GANTC motif among genes significantly misregulated (upregulated or downregulated) (**A**), significantly downregulated (**B**) and significantly upregulated (**C**) in the Δ*ccrM* strain compared with the wild-type strain. (**D**) Frequency, relative to the entire genome, of genes whose promoter region (200 bp upstream the translational start codon) contains a GANTC in one or two or in seven bacterial species closely related to *C. crescentus* (*C. crescentus*, *Caulobacter segnis*, *Caulobacter K31*, *Phenylobacter zucineum*, *Brevundimonas subvibrioides*, *Asticcacaulis excentricus*, *Maricaulis maris*) among genes significantly downregulated in the Δ*ccrM* strain. Stars indicate that the bias is significant (*P* < 0.05, Fisher’s exact test).

A subset of the transcriptional effects observed in the Δ*ccrM* strain was probably directly due to the absence of methylation at promoter regions containing GANTC motifs (140 misregulated genes contained minimum one GANTC motif in their promoter region). To assess whether the transcriptome analysis was likely to reveal such direct effects of GANTC methylation on transcription, we calculated the probability that significantly misregulated genes contained a GANTC motif in their promoter region. Because many cell cycle–regulated genes are controlled by relatively long promoter regions in *C. crescentus* (67), we looked for the presence of GANTC motifs up to 200 bp upstream of the annotated translational start codon of each gene. We found that the probability to find a GANTC motif was ∼1.8-fold higher than in a random set of genes (Fisher’s exact test *P* < 0.05) (Figure 5A). This value rose to ∼2.5-fold when considering only the genes misregulated >2-fold (Fisher’s exact test *P* < 0.05). There was a clear enrichment in genes containing a GANTC motif in their promoter region among the genes that were the most significantly up- or downregulated in the Δ*ccrM* strain (Figure 5B and C). The conservation of a GANTC motif in the promoter regions of homologous genes in closely related bacteria has been previously used as an indicator that the methylation of the motif may play a role in regulating the activity of the promoter (36). We thus asked whether genes strongly misregulated in the Δ*ccrM* strain had a higher probability of containing a ‘conserved’ GANTC motif, compared with a random set of genes. We found that genes containing a conserved GANTC motif were more frequent among the misregulated genes than expected randomly. Genes containing a GANTC motif that was conserved in seven closely related species were, for example, five times more frequent among the most downregulated genes than expected randomly (Fisher’s exact test *P* < 0.05) (Figure 5D). This result indicates that promoters that contain conserved GANTC motifs are more likely to be affected by the absence of CcrM. Thus, we predict that genes that are significantly misregulated in the Δ*ccrM* strain and that are under the control of a promoter region that contains a conserved GANTC motif have a high probability of being directly regulated by the methylation state of their promoter region (Supplementary Table S4). The *ctrA*, *ftsZ* and *mipZ* genes, which were shown to be directly regulated by DNA methylation (35,36), fit these criteria (Table 1). Additional interesting candidates from our study include, for example, *CCNA_01450* (*recJ*), *CCNA_01651* (*gyrA*), *CCNA_02283* (annotated as an endonuclease), *CCNA_02389* (UDP-N-acetylglucosamine pyrophosphorylase) and *CCNA_02726* (annotated as an acetyltransferase) (Supplementary Table S4).

**Figure 6.**
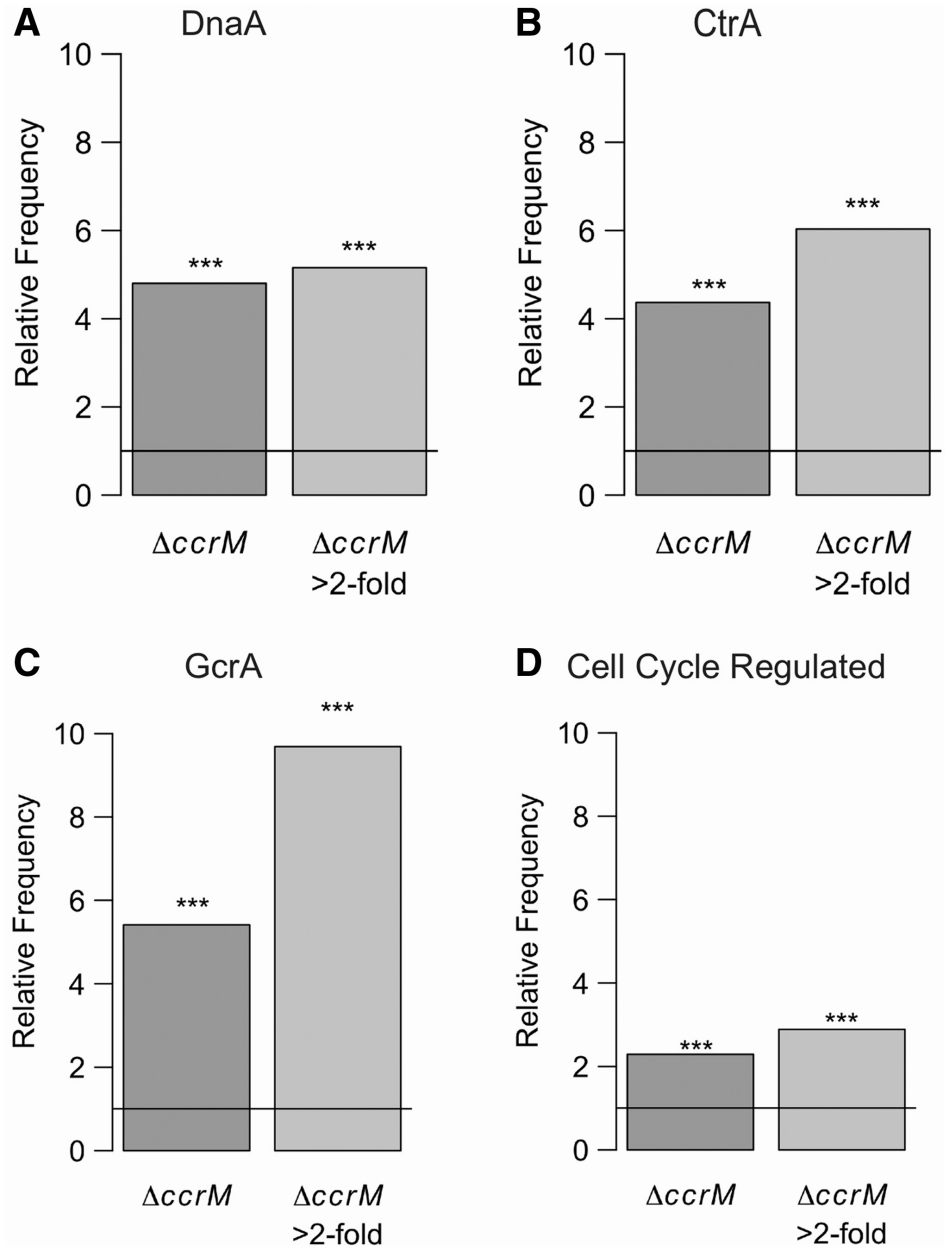
Involvement of CcrM in the regulation of the *C. crescentus* cell cycle. Frequency, relative to the entire genome, of genes belonging to the direct DnaA regulon (69) (**A**), the direct CtrA regulon (68) (**B**), the GcrA regulon (70) (**C**) or whose expression levels vary during the cell cycle (71) (**D**) among genes significantly downregulated or strongly downregulated (>2-fold change) in the Δ*ccrM* strain. Stars indicate a significant bias (*P* < 0.05, Fisher’s exact test). A comparison between genes significantly misregulated in the Δ*ccrM* strain and possible direct targets of GcrA, as determined by a ChIP-seq experiment (72), is shown in Supplementary Figure S10.

To evaluate the major physiological consequences of the transcriptional changes observed in the absence of CcrM-mediated methylation, we first examined the set of genes that were reported to be essential for the viability of *C. crescentus* (67) and that were significantly (corrected Student’s test *P* < 0.01) downregulated: we found that 31 essential genes (Supplementary Table S4), including four genes encoding cell division proteins (FtsZ, MipZ, FtsN and FtsE; *ftsX*, encoding FtsX, is co-transcribed with *ftsE* and misregulated at *P* < 0.05) and seven genes encoding proteins involved in DNA replication or repair or in the maintenance of chromosome topology (Ssb, RecJ, ligase, GyrA, Topo IV, thymidylate synthase and NrdA). Each of these 18 essential genes contained a relatively conserved GANTC motif in their promoter region (Table 1), suggesting that their expression might be directly activated by CcrM-mediated DNA methylation. We also observed that genes belonging to the GcrA (70), DnaA (69) and CtrA (68) regulons were strongly enriched among the most significantly downregulated genes in the Δ*ccrM* strain (Figures 6A–C), with the GcrA regulon being the most striking case (∼10-fold enrichment when considering genes whose transcription is changed at least 2-fold in the Δ*ccrM* strain). Genes whose expression is temporally regulated during the cell cycle (71) were also significantly enriched (Figure 6D). Altogether, these observations indicate a strong connection between DNA methylation by CcrM and cell cycle control, considering that GcrA, DnaA and CtrA are essential master transcriptional regulators of the *C. crescentus* cell cycle and that many essential genes might be directly regulated by DNA methylation (Table 1).

### Effect of the constitutive over-expression of CcrM on global transcription profiles in *C. crescentus*

To evaluate the global impact of CcrM-dependent methylation on gene expression in *C. crescentus*, we also analyzed the transcriptional profiles of bacterial populations in which CcrM is expressed at all times in the cell cycle. When CcrM is over-expressed, the period of post-replicational hemi-methylation of GANTC motifs that is characteristic of the wild-type strain is abolished having potential consequences on gene expression (Figure 3). We found that the expression levels of 546 genes were significantly changed in the strain with constitutive over-expression of CcrM compared with the wild-type strain, with 214 of these genes being affected >2-fold (Supplementary Table S5). For 15 genes, the effects were verified by qRT-PCR and were essentially consistent with the microarray results (Supplementary Figure S6).

To evaluate whether these effects may result from direct effects of GANTC methylation on promoter activity, we calculated the probability that significantly misregulated genes contained a GANTC motif in their promoter region and compared this value with the probability obtained for a random set of genes. We found no significant (Fisher’s test *P* < 0.05) difference, even when considering the most misregulated genes (Supplementary Figure S7), which suggests that most of the changes in gene expression observed in cells over-expressing CcrM result from indirect, rather than direct, effects of GANTC methylation on the activity of promoters.

**Figure 7.**
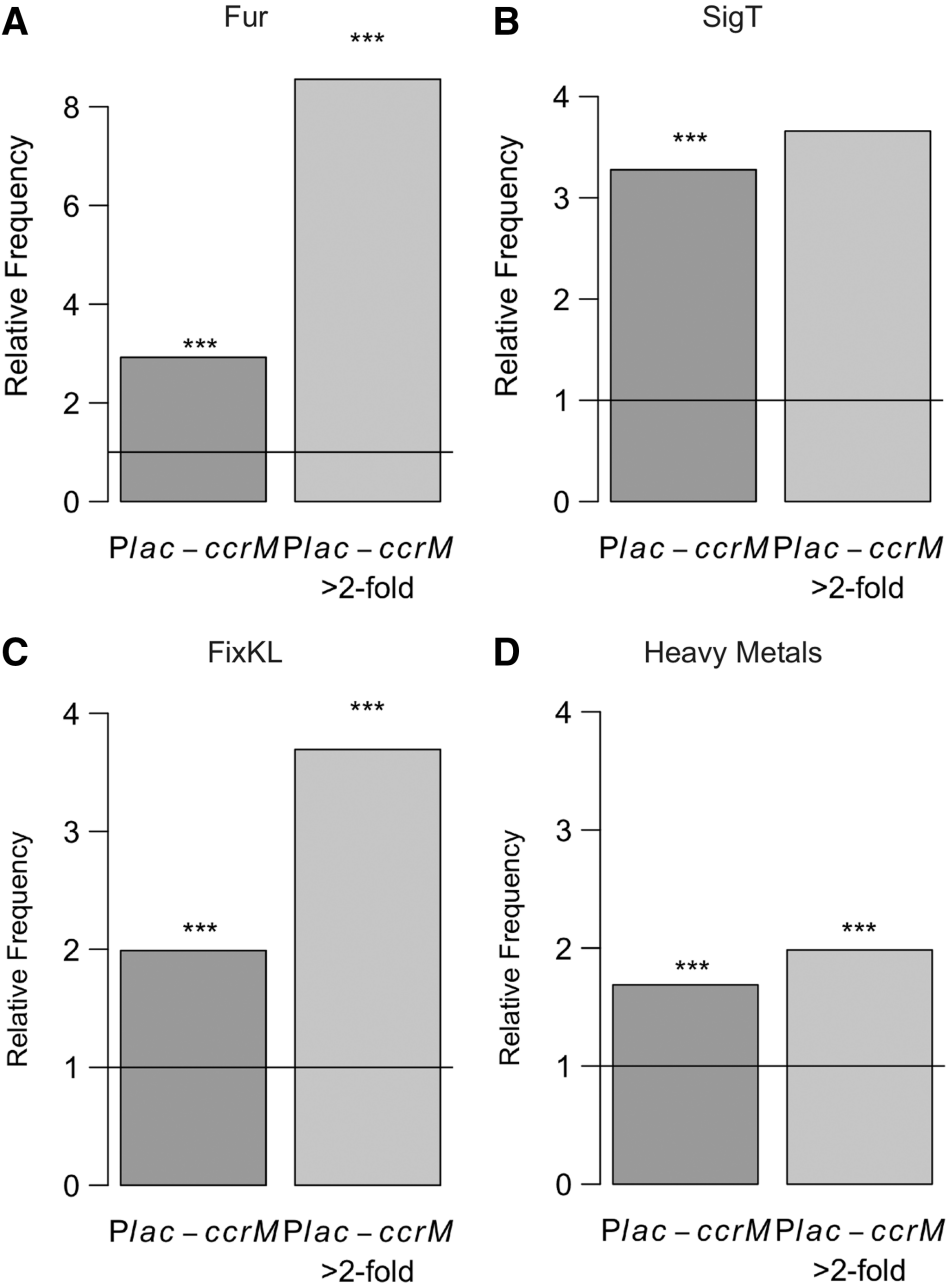
Constitutive over-expression of CcrM yields a stress phenotype. Frequency, relative to the entire genome, of genes belonging to the Fur regulon (**A**) or the SigT regulon (**B**), among genes significantly misregulated (upregulated or downregulated) or strongly misregulated (>2-fold change) in the P*lac::ccrM* strain. Frequency, relative to the entire genome, of genes belonging to the FixKL regulon (**C**) or the set of genes induced under heavy metal stress (**D**), among genes significantly misregulated or strongly misregulated (>2-fold change) in the P*lac::ccrM* strain. Stars indicate a significant bias (*P* < 0.05, Fisher’s exact test).

Only a few genes involved in the regulation of the cell cycle were found among the genes that were significantly misregulated in the CcrM over-expressing strain. Instead, genes encoding proteins involved in inorganic ion transport and metabolism were over-represented among the most downregulated genes in this mutant strain, while genes encoding proteins involved in DNA replication, recombination and repair were over-represented among the most upregulated genes (Figure 4B). Notably, regulons involved in the resistance to different forms of environmental stress, such as the Fur (73), SigT (74) and FixKL (75) regulons, were over-represented among misregulated genes (Figure 7A–C). Similarly, genes upregulated under heavy-metal stress (76) were also over-represented (Figure 7D). These observations suggest that cells with over-expressed CcrM throughout the cell cycle exhibit a general and unspecific stress response.

## DISCUSSION

**Figure 8.**
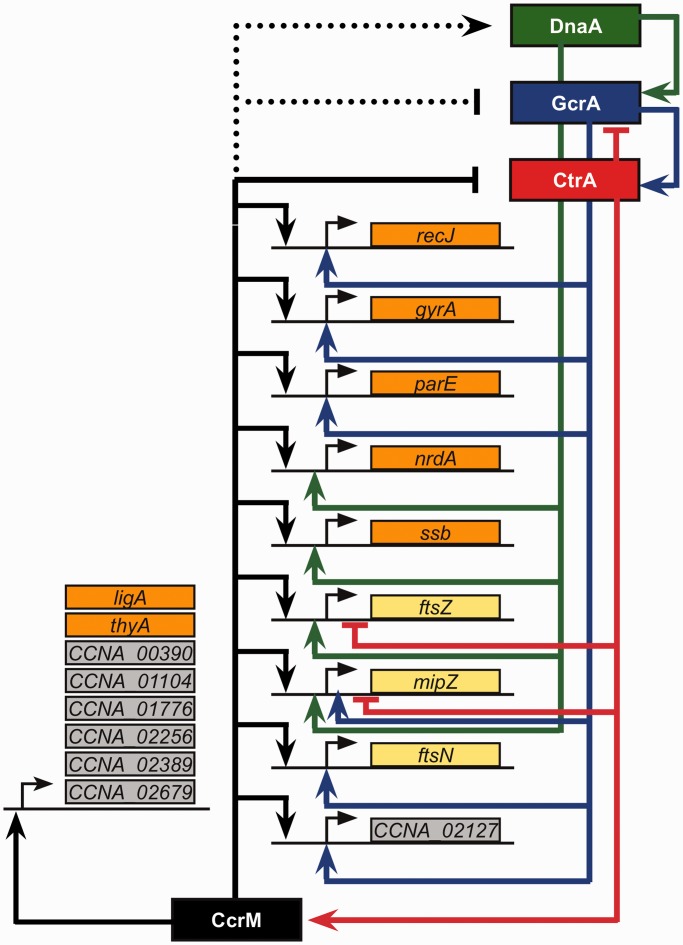
Model for the CcrM-dependent activation of essential genes containing conserved GANTC motifs in their promoter. Genes included in this schematic contain a GANTC motif in their promoter region (200 bp upstream of their translational start codon), which is conserved in a minimum of two out of five bacterial species closely related to *C. crescentus* (Table 1). Genes coloured in orange encode proteins involved in DNA replication, repair or topology. Genes coloured in yellow encode proteins involved in cell division. Genes coloured in grey encode proteins involved in other functions. Solid black arrows indicate regulatory pathways identified as significant during the transcriptome analysis using the Δ*ccrM* strain in this study. Dashed black arrows indicate regulatory effects previously identified (34,77), but not found as significantly affected using the Δ*ccrM* strain in this study. Solid blue, red and green arrows indicate the GcrA-, CtrA- and DnaA-dependent regulatory pathways identified in (70), (68) and (69), respectively. This schematic suggests that many, but not all, essential genes activated by CcrM are also co-regulated by DnaA, CtrA or GcrA master regulators, showing the strong interconnection between CcrM-dependent DNA methylation and the *C. crescentus* cell cycle regulatory network.

In the present study, we analyzed the methylation state of all GANTC motifs on the chromosome of three *C. crescentus* strains expressing different levels of the CcrM DNA methyltransferase (Figure 3) and determined the global transcriptional changes associated with the differences in the methylation state. We observed that hundreds of genes were misregulated in cells lacking CcrM or in cells in which CcrM is constitutively over-expressed throughout the cell cycle, compared with wild-type cells (Supplementary Tables S4 and S5). A significant proportion of the gene expression changes detected in the CcrM-deficient strain appeared to be due to direct methylation-mediated gene regulation, since conserved GANTC motifs were more frequently found in the promoter regions of genes misregulated in this strain than in random promoter regions (Figure 5 and Supplementary Table S4). Considering that 80 genes with a GANTC motif in their promoter region are found among genes strongly misregulated in the Δ*ccrM* strain and that this number is 2-fold higher than expected by chance, we estimate that ∼40 of these genes could be directly regulated by CcrM (some essential candidates are shown in Table 1 and Figure 8). In contrast, gene expression changes detected in the CcrM over-expressing strain appeared to be mostly due to secondary effects of the constitutive methylation of the chromosome that yields a stress phenotype (Figure 7 and Supplementary Figure S7). Consistent with these findings, we found no negative correlation between the changes in gene expression observed in Δ*ccrM* cells and in CcrM-over-expressing cells for most genes (Supplementary Figure S8), as would have been expected if the absence of methylation and constitutive-methylation had detectable opposite effects on the activity of many promoters. We also observed that genes misregulated in each strain generally encoded proteins involved in different processes (Figure 4).

In the absence of CcrM-dependent methylation, multiple genes involved in processes that are essential to the viability of *C. crescentus* were misregulated (Table 1 and Supplementary Tables S4). Figure 8 shows 17 essential genes that are good candidates for being directly activated by CcrM-dependent methylation, as they have a relatively conserved GANTC motif in their promoter region. These genes include many genes encoding proteins required for cell division, such as FtsZ, FtsN and MipZ, and proteins involved in DNA replication, repair and topology, such as the ligase, the gyrase and Ssb (Figure 8, Table 1 and Supplementary Table S4). We previously showed that the artificial expression of FtsZ can restore the viability of Δ*ccrM* cells in fast-growing conditions, but that these cells were still longer, straighter and with shorter stalks than wild-type cells (36). Our transcriptome analysis suggests that these cells might be longer due to insufficient quantities of FtsN or to excessive quantities of FtsW (Supplementary Table S4: the *ftsN* and *ftsW* messengers were 2.1 times less and 1.6 times more abundant in Δ*ccrM* than in wild-type cells, respectively). Interestingly, Δ*ccrM* cells still seemed to replicate their chromosome efficiently and had a normal spontaneous mutation rate (Supplementary Figures S4 and S5), despite the downregulation of several genes involved in DNA replication or repair and the over-expression of the CtrA inhibitor of replication (Figure 8 and Table 1 and Supplementary Table S4). This result suggests that proteins like Ssb or the ligase are not in limiting quantities for DNA replication and that the post-transcriptional regulation of CtrA maintains intracellular levels of active CtrA low enough for DNA replication to initiate (29). Consistent with our results, it was previously shown that a *C. crescentus* strain that carries a unique copy of the *ctrA* gene under the control of a mutant un-methylatable *ctrA* promoter does not have an abnormal phenotype (35). In addition to these many essential genes, several genes involved in processes that are required for the development of *C. crescentus* were also misregulated in the absence of CcrM-dependent methylation. For example, the *staR* and *creS* non-essential genes, involved in stalk biogenesis (78) and in cell curvature (79), respectively, were significantly downregulated in Δ*ccrM* cells (Supplementary Table S4: the *staR* and *creS* messengers were 2.9 and 1.6 times less abundant in Δ*ccrM* than in wild-type cells, respectively) and this might provide an explanation for the additional phenotypes that were observed.

Another remarkable finding was that genes regulated by the three global transcriptional regulators of the *C. crescentus* cell cycle, GcrA, CtrA and DnaA, were strongly over-represented in the set of genes that were the most misregulated in the absence of CcrM-mediated methylation (Figure 6 and Supplementary Figure S9 and S10). These three regulators are essential to the viability of *C. crescentus*, and they regulate the expression of hundreds of genes involved in various events of the cell cycle (27). Our observation suggests that *C. crescentus* genes encoding proteins that are critical for cell cycle progression are often regulated by one or more global regulators and by DNA methylation (Figures 6 and 8 and Table 1), showing the strong interconnection between CcrM-dependent DNA methylation and the *C. crescentus* cell cycle regulatory network. A possible explanation is that the binding or the activity of one or more of these global regulators is sensitive to the methylation state of the DNA at or next to their binding site. The consensus binding sites of CtrA and DnaA do not contain a sequence that resembles a GANTC motif (68,69). As for GcrA, its consensus binding site is not yet clearly characterized, but a recent study demonstrated that GcrA activates the transcription of several genes by promoting the recruitment of the RNA polymerase to promoter regions containing methylated GANTC motifs that overlapped a GcrA binding site (72). Interestingly, the homologs of GcrA have the same conservation pattern as CcrM homologs (Figure 2A). The expression of many other members of the GcrA direct regulon (68,72) was nevertheless not significantly affected in the CcrM-deficient strain (Supplementary Table S4 and Supplementary Figure S9), suggesting that the activity of GcrA might not always be influenced by CcrM-dependent methylation. Further studies will be required to test whether the activities of the CtrA and DnaA global regulators may also be influenced by the methylation state of some promoter regions or if the over-representation of the CtrA and DnaA regulons among genes significantly misregulated in the Δ*ccrM* strain is only a consequence of regulation by more than one regulator.

The chromosomes of cells over-expressing CcrM are maintained in the fully methylated state throughout the cell cycle (Figure 3); these cells are slightly elongated and tend to accumulate additional copies of the chromosome (31). The microarray analyses revealed that the transcription levels of many genes are changed in this mutant (Supplementary Table S5) and that cells exhibit a stress response (Figure 7). Transcriptional effects directly due to the elimination of the fully methylated to hemi-methylated switch characteristic of the wild-type strain were, however, either rare or so weak that the method was not sensitive enough to detect them (Supplementary Figure S7). A possible link between the over-replication phenotype of the strain and the transcriptome can be found in the downregulation of the gene encoding the PopZ protein (Supplementary Table S5: the *popZ* messenger was 1.4 times less abundant in CcrM-over-expressing cells than in wild-type cells), which is required for the anchoring of the two chromosomal origins at opposite cell poles after the initiation of chromosome replication (80,81). Insufficient intracellular levels of PopZ may contribute to the abnormal DNA content in cells over-expressing CcrM, as was previously described for PopZ-depleted cells (80,81). Other phenotypes may be attributable to a global stress response that seems to take place in these cells (Figure 7).

Our SMRT sequencing analysis showed that 35 GANTC motifs along the chromosome were frequently under-methylated on minimum one strand in wild-type cells (Supplementary Table S3). We found that only three of these motifs remained under-methylated in cells over-expressing CcrM. To address whether the under-methylation of the other 32 GANTC motifs in wild-type cells may affect gene expression, we compared the mRNA levels of nearby genes in the wild-type and in the CcrM-over-expressing cells. We observed a significant difference in expression of two genes: these were the *CCNA_01150* gene of unknown function and the *CCNA_03248* gene encoding a TonB-dependent receptor (Supplementary Tables S3 and S5): the *CCNA_01150* and the *CCNA_03248* messengers were five times more and 1.3 times less abundant in CcrM-over-expressing than in wild-type cells, respectively). The difference in expression might be linked to the difference in the methylation state of GANTC motifs in the two strains. Similarly, under-methylated GATC sites were observed on the chromosomes of Dam-expressing bacteria and they are sometimes involved in epigenetic switches of gene expression (7,11,24–26,82). These sites often overlap the binding sites of the Lrp, OxyR and Fur global regulators, which were shown to compete with Dam for the DNA and to be sometimes sensitive to its methylation state. Interestingly, we found that the predicted Fur regulon was over-represented among the genes that were misregulated when the CcrM enzyme was over-produced in *C. crescentus* (Figure 7). We nevertheless did not find Fur-regulated promoters carrying frequently under-methylated GANTC motifs on the *C. crescentus* chromosome. This observation suggests that the *C. crescentus* Fur protein does not often protect the promoter that it controls from CcrM-mediated methylation, at least when cells were cultivated in iron-rich medium, contrarily to what was previously observed at the *sci1* promoter on the *E. coli* chromosome (82). It is still possible that CcrM-dependent methylation affects the activity of the *C. crescentus* Fur protein using a mechanism different from the one previously described in *E. coli* (82). An excellent candidate gene that may be regulated by an epigenetic switch mediated by Fur and DNA methylation in *C. crescentus* is the *CCNA_02275* gene, as its promoter contains two GANTC sites that overlap a Fur binding site and since its messenger was 2.2 times less abundant in CcrM-over-expressing than in wild-type cells (Supplementary Table S5). This gene encodes a trans-membrane protein of unknown function and is repressed by Fur (83). These results suggest that Fur may have a higher affinity for the *CCNA_02275* promoter when it is in a fully methylated, rather than in a hemi-methylated, state. Additional detailed studies will be required to understand how this gene might be epigenetically regulated in *C. crescentus*.

Overall, our study demonstrates that the overlap between the roles of Dam-dependent methylation in enterobacteria and the roles of CcrM-dependent methylation in *Alphaproteobacteria* is restricted. In both cases, the methylation of adenines has pleiotropic effects on gene expression, but the genes and functions represented in the Dam and CcrM regulons are different. In *C. crescentus*, but not *E. coli*, the methylation of GANTC motifs may mediate the integration of the cell cycle regulatory circuit with chromosome replication. In addition, we found that CcrM-dependent methylation is not required to control the initiation of chromosome replication (Supplementary Figure S4) or to correct DNA mismatches during the MMR process (Supplementary Figure S5), as it is often the case for Dam-dependent methylation (11,12,14). Dam is generally co-conserved with MutH, which is the protein that recognizes the newly synthesized non-methylated DNA strand that needs to be repaired during methyl-directed MMR (18). Most bacteria lack Dam and MutH, like *C. crescentus* and *Bacillus subtilis* (13,84,85). These bacteria probably use a methylation-independent strand recognition mechanism during MMR, which might be spatially coupled with the DNA polymerase complex (85). This study exemplifies the need to explore the multifaceted use of DNA methylation in a variety of bacterial species.

## ACCESSION NUMBERS

The microarray data are publicly available at the GEO database (GEO accession numbers GSE52722 and GSE52375, https://www.ncbi.nlm.nih.gov/geo).

## SUPPLEMENTARY DATA

Supplementary Data are available at NAR Online.

## FUNDING

University of Lausanne; Swiss National Science Fellowships [3100A0_122541 and 31003A_140758 to J.C.]; National Institute of Health Grant [RO1-GM051426 to L.S.]. Funding for open access charge: Swiss National Science Fellowship [31003A_140758 to J.C.].

*Conflict of interest statement*. None declared.

## Supplementary Material

Supplementary Data
