# Supplementary material for: The functions of DNA methylation by CcrM in Caulobacter crescentus: a global approach
Source: Nucleic Acids Res. 2014 Jan 7;42(6):3720–35. doi: 10.1093/nar/gkt1352 (PMC3973325; doi:10.1093/nar/gkt1352)
Supplement: Supplementary Data [file supp_42_6_3720__index.html]

The functions of DNA methylation by CcrM in Caulobacter crescentus: a global approach — The functions of DNA methylation by CcrM in Caulobacter crescentus: a global approach — Supplementary Data 

# The functions of DNA methylation by CcrM in *Caulobacter crescentus*: a global approach

## Supplementary Data

files

**Files in this Data Supplement:**

- Supplementary Data - zip file
