# Supplementary material for: The functions of DNA methylation by CcrM in Caulobacter crescentus: a global approach
Source: Nucleic Acids Res. 2014 Jan 7;42(6):3720–35. doi: 10.1093/nar/gkt1352 (PMC3973325; doi:10.1093/nar/gkt1352)
Supplement: Supplementary Data [file supp_gkt1352_suppl_data.zip › nar-02997-h-2013-File010.pdf]

<u>**SUPPLEMENTARY INFORMATION**</u>

The functions of DNA methylation by CcrM in *Caulobacter crescentus:* a global approach

Diego Gonzalez, Jennifer B. Kozdon,
Harley H. McAdams, Lucy Shapiro and Justine Collier

SUPPLEMENTARY FIGURES:

A

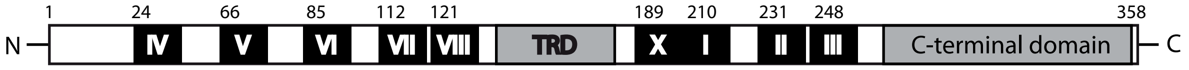

B

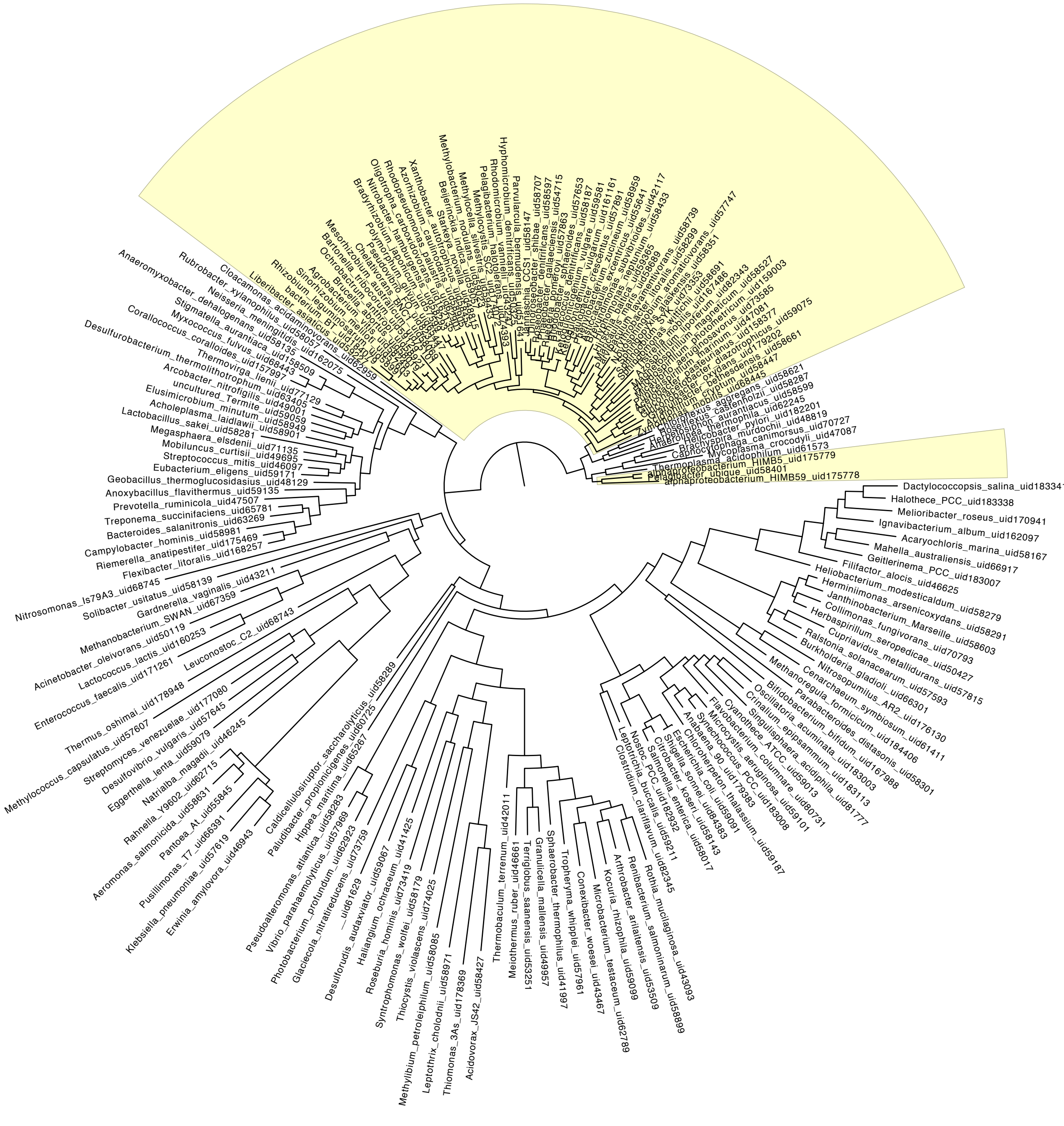

C

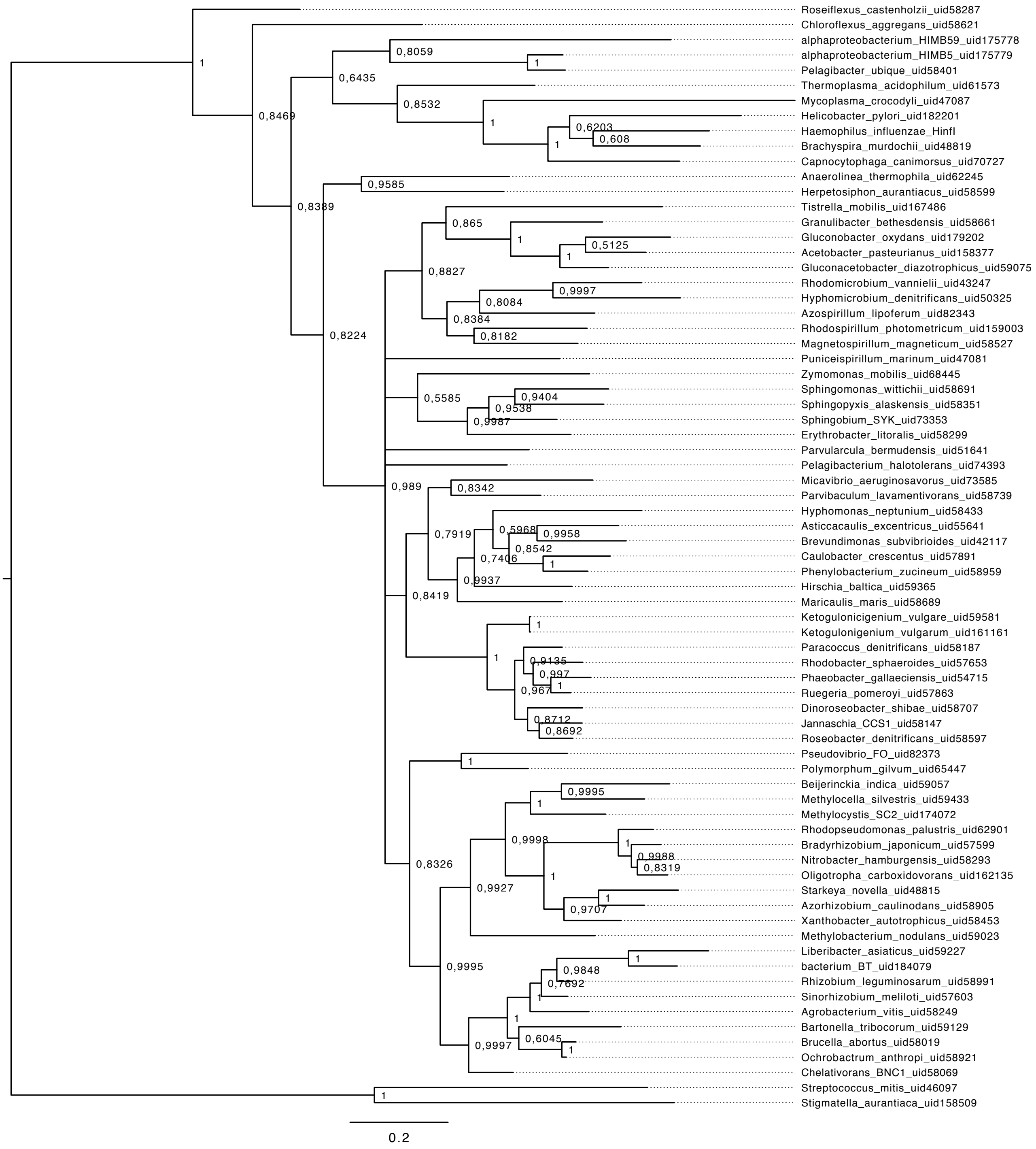

Figure S1: **Conservation of CcrM-like DNA methyltransferases in bacteria.** (A) Structure of the group of CcrM/HinfI-like adenine N6-methyltransferases. All 9 motifs characteristic of DNA amino-methyltransferases (motifs IV, V, VI, VII, VIII, X, I, II, III) can be found in CcrM homologs in the expected order for the β class of DNA amino-methyltransferases (86). An additional C-terminal domain is found in all *Alphaproteobacterial* CcrM homologs and only a few methyltransferases outside *Alphaproteobacteria* (among which is M.HinfI from *Haemophilus influenzae*). TRD stands for "Target Recognition Domain", i.e. the DNA binding domain specific for the recognition site of the DNA methyltransferase. (B) Phylogenetic tree of members of the β class of N6-adenine methyltransferases that show a significant homology with CcrM from *C. crescentus. Alphaproteobacteria* are highlighted in yellow. Protein sequences were aligned with MUSCLE and the tree was produced with FastTree using gamma-distributed rates of site evolution and the WAG substitution matrix. Support values

are shown at the nodes. (C) Phylogenetic tree produced with MrBayes from the set of members of the β class of N6-adenine methyltransferases that show a high homology with CcrM from *C. crescentus* and have a conserved C-terminal domain, with the addition of sequences from *Streptococcus mitis* and *Stigmatella aurantiaca* as outgroups. The branches with support values < 0.5 were left unresolved.

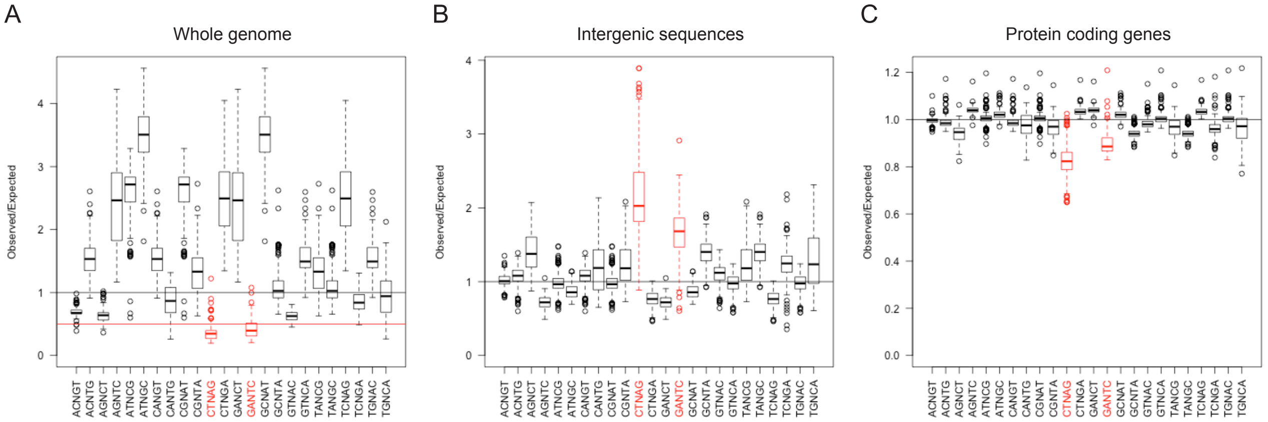

Figure S2: **Distribution of pentanucleotides of the same structure as GANTC in the genomes of non-*Rickettsiales Alphaproteobacteria*.** Observed over expected ratios for all quintuplets of nucleotides in total (A), intergenic (B) and genic (C) sequences in non-*Rickettsiales Alphaproteobacteria*. The expected number of motifs was calculated for each genome taking the nucleotide composition into account. The limits of the box represent the first and third quartiles and the bold line the median of the distribution. The particular distribution of GANTC and CTNAG motifs is shown in red. The red line indicates an observed/expected ratio of 0.5.

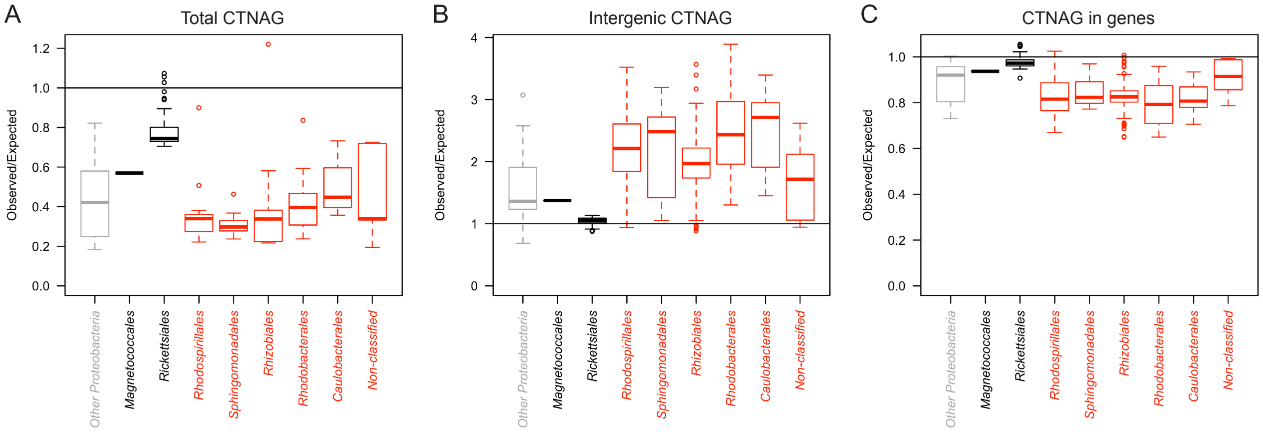

Figure S3: Distribution of CTNAG motifs in the genomes of different orders of *Alphaproteobacteria*. Observed over expected CTNAG ratios in the genomes (A), the intergenic sequences (B) and the coding sequences (C) of all sequenced species in the main orders of *Alphaproteobacteria* and a control of 10 mixed other *Proteobacteria*. The expected number of CTNAG motifs was calculated for each genome taking the nucleotide composition into account. The limits of the box represent the first and third quartile and the bold line the median of the distribution. The groups whose members have a CcrM homolog are in red.

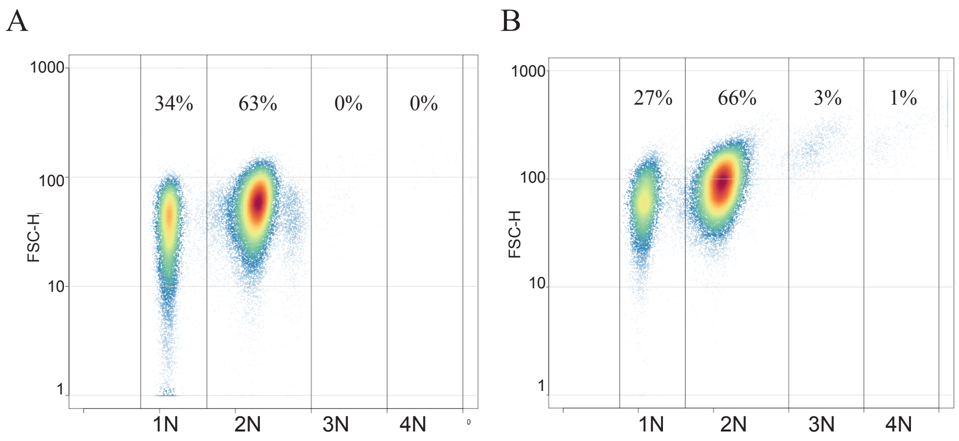

Figure S4: Chromosome replication and segregation do not seem to be affected in Δ*ccrM* cells. Composition of NA1000 (A) and Δ*ccrM* (B) populations cultivated in M2G to exponential phase, and collected after 6 hours of rifampicin treatment. The analysis was performed by flow cytometry; the parameters used to represent the data are FSC-H (forward scattering), which is a measure of the size of the cell, and the fluorescence (FL2) that quantifies the chromosome content. 1N, 2N, etc. represent the number of complete chromosomes in the cells. The % of cells containing 1N, 2N, 3N or 4N chromosomes is indicated in each panel.

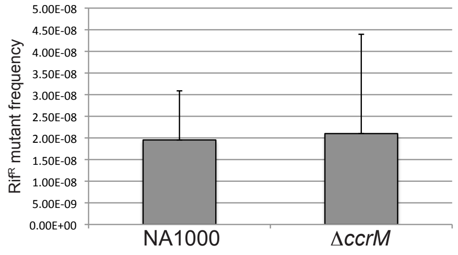

Figure S5: The spontaneous mutation rate is similar in Δ*ccrM* and in wild type cells. Frequency of Rifampicin resistant (Rif^R) mutants in a NA1000 and Δ*ccrM* populations cultivated in M2G to exponential phase. The error bars indicate standard deviations from three independent experiments.

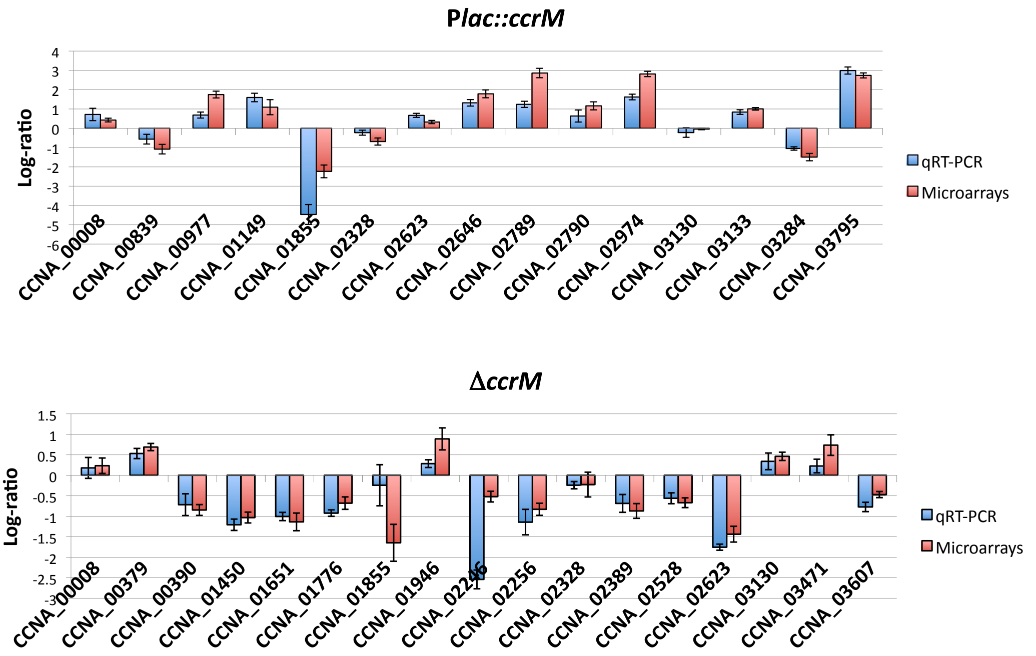

Figure S6: **qRT-PCR analyses essentially confirm microarray results.** Assessment of the expression change observed in the microarray data for selected genes based on qRT-PCR assays. Red bars represent the log-ratios obtained via microarrays, blue bars the corresponding log-ratios obtained by qRT-PCR. The results obtained by microarrays and qRT-PCR are mostly consistent in direction and amplitude. The major discrepancies were found in the Δ*ccrM* strain: the *CCNA_02246* (*mipZ*) gene is represented by a set of unspecific low-signal probes on the microarrays which probably accounts for the underestimation of the down-regulation in the Δ*ccrM* strain by microarrays; the difference between microarrays and qRT-PCR for the *CCNA_01855* (superoxide dismutase) gene expression is most likely biological and batch-dependent.

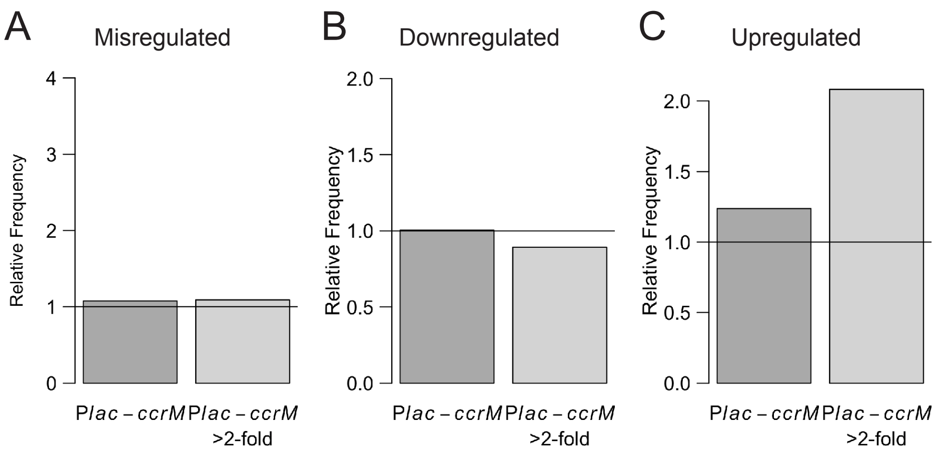

Figure S7: **GANTC motifs are not significantly enriched upstream of genes whose expression changed in CcrM-overexpressing cells.** Frequency, relative to the entire genome, of genes whose promoter region (200 bp upstream of their start codon) contains a GANTC site among genes significantly or strongly (more than two fold change) misregulated (A), significantly or strongly downregulated (B) and significantly or strongly upregulated (C) in the P*lac::ccrM* strain compared to the wild type strain. None of these biases were considered as significant (p-value < 0.05, Fisher's exact test).

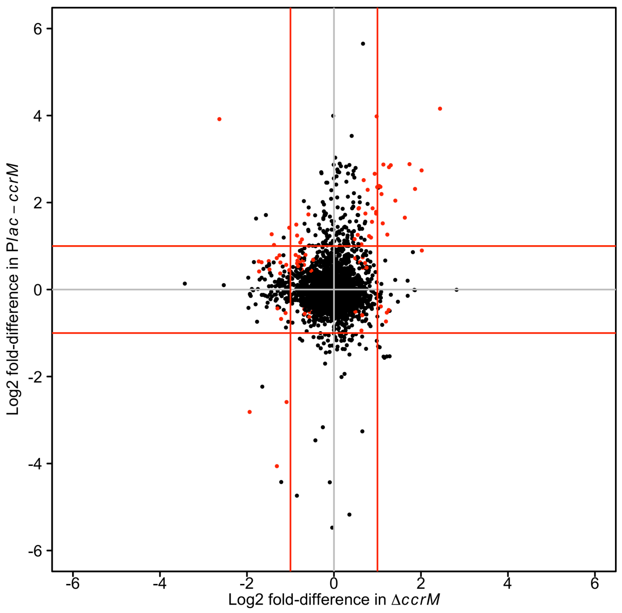

Figure S8: **Absence of correlation between the transcriptome analyses using strains expressing different levels of CcrM.** Microarray log-ratios (Δ*ccrM* or p*lac*::*ccrM* mutant vs NA1000) for all *C. crescentus* genes. Red lines indicate the 2-fold change limit. Red dots represent genes that are significantly misregulated in both Δ*ccrM* and P*lac::ccrM* strains. No inverse correlation of the log-ratios were observed overall or among genes significantly modified (p-value < 0.01, corrected Student's test) in both strains (cor = 0.079 and 0.014 respectively).

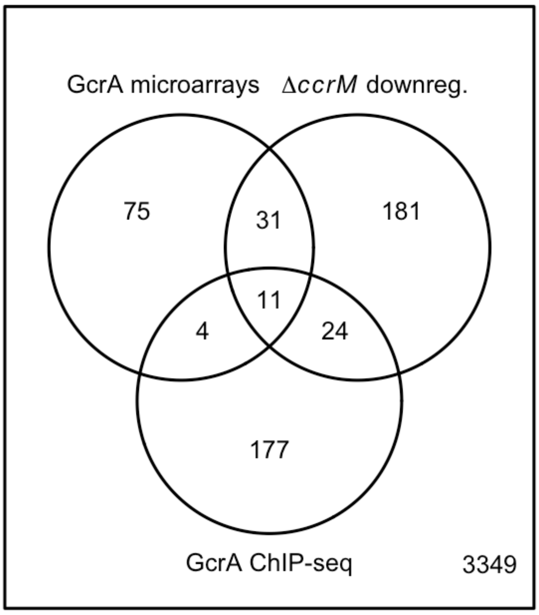

Figure S9: GcrA-regulated genes are not always misregulated in the Δ*ccrM* strain. Venn diagram showing the overlaps between the GcrA regulon defined by microarrays using the GcrA-depletion strain (68), the GcrA direct regulon defined by ChIP-Seq experiments using the wild type strain (78) and the genes that are significantly down-regulated in the Δ*ccrM* strain (this study).

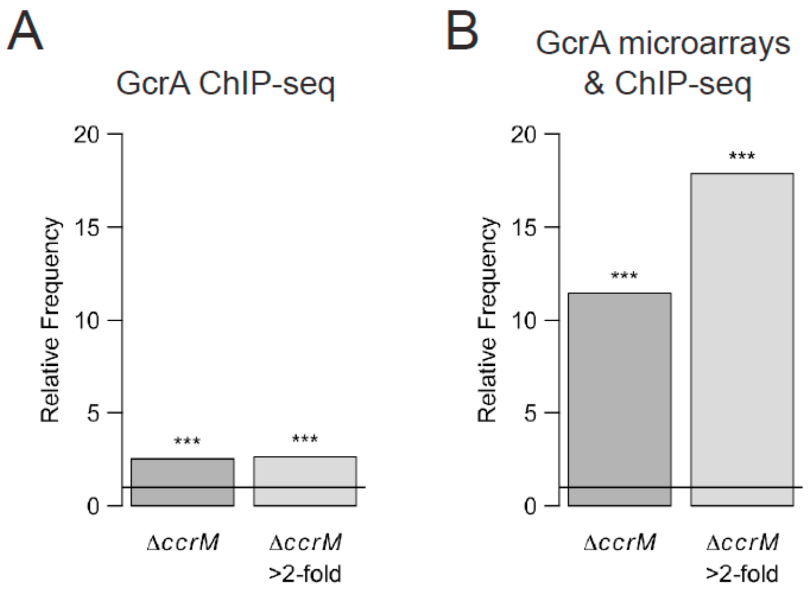

Figure S10: **The GcrA direct regulon is over-represented among genes that are the most misregulated in the Δ*ccrM* strain.** (A) Frequency, relative to the entire genome, of genes whose promoter region binds to GcrA (78) among genes significantly misregulated in the Δ*ccrM* strain. To determine the GcrA direct regulon using the ChIP-Seq data from (1), we selected genes in the promoter region of which a GcrA peak was observed; the promoter region was defined as the sequence from -200 bps to + 20 bps from the translational start codon. (B) Frequency, relative to the entire genome, of genes whose promoter region binds to GcrA (1) and whose expression is affected in a GcrA-depletion strain as determined by microarray experiments (2) among genes significantly misregulated in the Δ*ccrM* strain. Stars indicate a significant bias (p-value < 0.05, Fisher's exact test).

SUPPLEMENTARY TABLES FOOTNOTES:

Table S1: CcrM homologs in bacterial proteomes.

Table S2: Genomic context of the *ccrM* homologs.

Table S3: Lists of GANTC motifs with at least one adenine that is under-methylated in the genomes of the wild type or the P*lac*::*ccrM* strains.

Table S4: List of genes significantly misregulated in the Δ*ccrM* strain. This table also lists the genes that have a GANTC motif in their promoter region and the genes that are essential to the viability of *C. crescentus*.

Table S5: List of genes significantly misregulated in the P*lac*::*ccrM* strain. This table also lists the genes that have a GANTC motif in their promoter region and the genes that are essential to the viability of *C. crescentus*.

SUPPLEMENTARY MATERIALS AND METHODS :

Flow cytometry analysis

Flow cytometry analyses (Fig.S4) were performed as previously described (87). Cells were cultivated in exponential phase in M2G medium containing 15 μg/mL of rifampicin for 6 hours before sampling. Rifampicin blocks not only the initiation of DNA replication, but also cell division in *C. crescentus*, as there exists a checkpoint that links chromosome replication with cell division in

C. crescentus (88). The DNA of minimum 20000 cells from each biological sample was stained with Vybrant® DyeCycle™ Orange (Invitrogen). Data were collected using FL-2 fluorescence. Data were analyzed and visualized with R [using the "prada" package (Florian Hahne, Wolfgang Huber, Markus Ruschhaupt and Joern Toedling. Prada: data analysis for cell-based functional assays. R package version 1.24.0)]. The forward scattering (FSC-H) parameter was used to estimate cell sizes.

Determination of spontaneous mutation rates

Spontaneous mutation rates (Fig.S5) were measured using a rifampicin resistance assay as previously described (89). Cells were cultivated in exponential phase in M2G medium and serial dilutions were plated on M2G medium with or without 100 µg/mL rifampicin. Mutation frequencies were calculated by dividing the number of rifampicin-resistant colonies by the total number of colonies that grew on M2G.

Quantitative Real Time PCR

RNA samples were prepared as described in the Material and Methods section in the main text. 1 µl of each RNA sample was used as a template in 20 µl Rotor-Gene SybrGreen for a 40 cycle qRT-PCR analysis to ensure that DNA contamination was negligible. The other 10 µl of DNase I treated RNA samples were used as a template for the cDNA synthesis using the SuperScript II (Invitrogen) and random hexamers (Promega) in a 20 µl reaction mix (cycling: 12 min. at 25°C, 60 min. at 42°C, 15 min. at 70 °C); 0.5 µl RNase H (Invitrogen) was added and the samples were incubated for 20 minutes at 37°C. The cDNA was then diluted 1:20 in 10 mM Tris pH 8. A replicate of two samples was treated in the same way with the addition of H_2O instead of the SuperScript II enzyme ("No Enzyme Controls" [NECs]). A total of 11 samples (9 samples of cDNA and 2 NECs) were used as templates for qRT-PCR analysis.

Genes intended for qRT-PCR analysis were selected on the basis of their fold-change in the microarray results and on their biological interest; *dnaA*, *ctrA*, *gcrA*, *ftsZ* and *mipZ* levels were quantified in all 11 samples. *CCNA_00496, CCNA_03642, CCNA_00317, CCNA_03876* were used as reference genes and quantified in all 11 samples. All primer pairs were checked for specificity by a standard 40 cycle PCR before performing the qRT-PCR. For each sample-primer pair, three RT-PCR reactions were set-up (technical triplicates). The individual reaction contained 2 µl template in 10 µl total reaction with 0.3 µM primers (using 5 µl 2x SybrGreen master mix). The qRT-PCR run was performed on an Applied Biosystems 7900HT Sequence Detection System. The baseline was adjusted manually for each primer pair before generating the Ct values. The Ct values for all NECs reactions were >35 cycles.

The data analysis was performed with home-made perl scripts. The Ct values were averaged among technical replicates. The three reference genes with lowest M value (90), i.e. with lowest variance relative to the other reference genes, were determined : *CCNA_00496, CCNA_03642, CCNA_00317; CCNA_03876* diverged from the other genes only in the JC1149 strain. The geometric mean of the three selected reference genes was calculated for each sample [=Ref]. To calculate the Delta-Delta-Ct for a target gene X in one of the mutant strain samples *mut* (JC1149 or JC362), we used the following equation: $(Ct(X)_{mut} - Ct(Ref)_{mut})$-$(Ct(X)_{NA1000} - Ct(Ref)_{NA1000})$. The Delta-Delta-Ct was calculated for each sample-primer pair; the values were then averaged per strain and the standard deviation was calculated.

86. Malone, T., Blumenthal, R.M. and Cheng, X. (1995) Structure-guided analysis reveals nine sequence motifs conserved among DNA amino-methyltransferases, and suggests a catalytic mechanism for these enzymes. J Mol Biol, 253, 618-632.
87. Fernandez-Fernandez, C., Gonzalez, D. and Collier, J. (2011) Regulation of the Activity of the Dual-Function DnaA Protein in Caulobacter crescentus. PLoS One, 6, e26028.
88. Jenal, U., Stephens, C. (2002) The Caulobacter cell cycle: timing, spatial organization and checkpoints. Current Opinion in Microbiology, 5, 558-563.
89. Galhardo, R.S., Rocha, R.P., Marques, M.V. and Menck, C.F. (2005) An SOS-regulated operon involved in damage-inducible mutagenesis in Caulobacter crescentus. Nucleic Acids Res, 33, 2603-2614.
90. Vandesompele, J., De Preter, K., Pattyn, F., Poppe, B., Van Roy, N., De Paepe, A. and Speleman, F. (2002) Accurate normalization of real-time quantitative RT-PCR data by geometric averaging of multiple internal control genes. Genome Biol, 3, RESEARCH0034.
